# Genomic prediction in hybrid breeding: I. Optimizing the training set design

**DOI:** 10.1007/s00122-023-04413-y

**Published:** 2023-08-02

**Authors:** Albrecht E. Melchinger, Rohan Fernando, Christian Stricker, Chris-Carolin Schön, Hans-Jürgen Auinger

**Affiliations:** 1 Plant Breeding, TUM School of Life Sciences, Technical University of Munich, 85354 Freising, Germany; 2 Institute of Plant Breeding, Seed Science and Population Genetics, University of Hohenheim, 70599 Stuttgart, Germany; 3 Department of Animal Science, Iowa State University, Ames, IA 50011 USA

## Abstract

**Key message:**

Training sets produced by maximizing the number of parent lines, each involved in one cross, had the highest prediction accuracy for H0 hybrids, but lowest for H1 and H2 hybrids.

**Abstract:**

Genomic prediction holds great promise for hybrid breeding but optimum composition of the training set (TS) as determined by the number of parents (*n*_TS_) and crosses per parent (*c*) has received little attention. Our objective was to examine prediction accuracy ($r_{a}$) of GCA for lines used as parents of the TS (I1 lines) or not (I0 lines), and H0, H1 and H2 hybrids, comprising crosses of type I0 × I0, I1 × I0 and I1 × I1, respectively, as function of *n*_TS_ and *c*. In the theory, we developed estimates for $r_{a}$ of GBLUPs for hybrids: (i)$\hat{r}_{a}$ based on the expected prediction accuracy, and (ii) $\tilde{r}_{a}$ based on $r_{a}$ of GBLUPs of GCA and SCA effects. In the simulation part, hybrid populations were generated using molecular data from two experimental maize data sets. Additive and dominance effects of QTL borrowed from literature were used to simulate six scenarios of traits differing in the proportion (*τ*_SCA_ = 1%, 6%, 22%) of SCA variance in *σ*_*G*_^2^ and heritability (*h*^2^ = 0.4, 0.8). Values of $\tilde{r}_{a}$ and $\hat{r}_{a}$ closely agreed with $r_{a}$ for hybrids. For given size *N*_TS_ = *n*_TS_ × *c* of TS, $r_{a}$ of H0 hybrids and GCA of I0 lines was highest for *c* = 1. Conversely, for GCA of I1 lines and H1 and H2 hybrids, *c* = 1 yielded lowest $r_{a}$ with concordant results across all scenarios for both data sets. In view of these opposite trends, the optimum choice of *c* for maximizing selection response across all types of hybrids depends on the size and resources of the breeding program.

**Supplementary Information:**

The online version contains supplementary material available at 10.1007/s00122-023-04413-y.

## Introduction

Genomic prediction has a huge potential for improving the efficiency of hybrid breeding as demonstrated by numerous studies with various allogamous crops such as maize, sunflower, rye, sugar beet, autogamous crops such as wheat, barley, triticale and partially allogamous crops such as oilseed rape (see Seye et al. (2020) for a recent review). The main reason is that the number of potential hybrids, which can be predicted using genomic data from their parents, is determined by multiplying the number of parents in each heterotic group. Consequently, the size of the prediction set (PS) increases in quadratic terms with the number of parents and allows to apply extremely high selection intensities (Westhues et al. 2017). Besides the optimum allocation of resources to be spent on phenotyping hybrids in the training set (TS) versus genotyping parent lines, the composition of the TS as determined by the number of parents and number of crosses per parent has a strong influence on the prediction accuracy ($r_{a}$) of the various types of hybrids in the PS (Technow et al. 2014; Seye et al. 2020). A parent of a hybrid in the PS is classified as an I1 line, if it has one or more hybrids in the TS; all other parents having no hybrid in the TS are classified as I0 lines. Thus, the PS is composed of H0, H1 and H2 hybrids, corresponding to crosses of type I0 × I0, I0 × I1 or I1 × I0, and I1 × I1 lines. Optimal integration of genomic selection in hybrid breeding requires detailed knowledge, how prediction accuracy of these different types of hybrids depends on the population structure of TS (Kadam et al. 2021). This applies not only to genomic but also to pedigree data (Bernardo 1996) and other types of “omics” data suggested in the literature for hybrid prediction including transcriptomic (Westhues et al. 2017; Zenke‐Philippi et al. 2017; Seifert et al. 2018), and metabolomic data (Riedelsheimer et al. 2012; Westhues et al. 2017) or combinations of them (Schrag et al. 2018).

In principle, two approaches exist for organizing the TS for genomic prediction in hybrid breeding. In the “testcross” approach, a separate TS is generated for each parent population by crossing candidate lines with the same tester(s) from the opposite population and testcross performance is used as a proxy for the GCA of the lines. In the “factorial” approach, the TS consists of inter-population hybrids between candidates from each parent population produced according to an incomplete factorial mating design. Both approaches were compared with simulations (Seye et al. 2020) and experiments (Fristche-Neto et al. 2018; Lorenzi et al. 2022). Since these studies demonstrated the superiority of sparse factorial designs for hybrid prediction and reciprocal recurrent genomic selection, we focused our investigations on this approach, with a main focus on the prediction accuracy of H0, H1, and H2 hybrids, as well as the GCA of I0 and I1 lines.

Hybrid performance is the main criterion for cultivar development, while general combining ability (GCA) of the parent lines is the main criterion for selecting the most promising parents for generating the base material for the next breeding cycle (Hallauer et al. 2010). In general, testcross performance with a genetically narrow tester (i.e., inbred line or single cross) from the opposite population is used as a proxy for the GCA of the candidate lines (Albrecht et al. 2011; Lian et al. 2014; Auinger et al. 2021), but these predictions are confounded with specific combining ability (SCA) effects of the candidates with the tester. In particular, a link between the prediction accuracy of hybrids and the prediction accuracy of their SCA and GCA of their parents is missing. A deeper understanding of the relationship between these components could help integrating product development with recurrent improvement of the parent populations in a genomic-based comprehensive approach to hybrid breeding.

Investigations on the optimum composition of the TS for hybrid breeding have been based on the prediction error variance (Fristche-Neto et al. 2018) or the CDmean criterion (Kadam et al. 2021). The genomic relationship matrix required for these approaches was calculated treating the hybrid population itself as reference base. However, this ignores the fact that unlike in other breeding categories in plant breeding (e.g., line, clonal and population breeding), the two parents of a hybrid usually originate from two genetically diverse populations for optimum exploitation of heterosis (Melchinger and Gumber 1998). Using a model with GCA and SCA effects, Seye et al. (2020) investigated the optimum composition of the TS with simulations. They analyzed a hybrid population consisting of several families. Each family was composed of inter-population hybrids that were produced from parent lines derived from diallel crosses of four founder lines in each parent population. The authors based their comparisons on prediction accuracies calculated across all families of hybrids but it is unknown to what extent the variance among subgroup means affected the prediction accuracy of H0, H1 and H2 hybrids within families, which is of main interest to the breeder.

The magnitude of the prediction accuracy is of fundamental importance for the optimum design of the TS. Estimating the prediction accuracy for sets of H0, H1 and H2 hybrids by cross-validation is hardly feasible in hybrid breeding, because this would require a huge TS, exceeding by far the capacity of most breeding programs. Alternative to cross-validation, $r_{a}$ can be estimated by an approximation of the expected prediction accuracy ($\hat{r}_{a}$) calculated from population parameters as described by Ould Estaghvirou et al. (2013). However, application of $\hat{r}_{a}$ to determine the prediction of H0, H1 and H2 hybrids under a GCA–SCA model has not been described in the literature hitherto.

Selection index formulas have been used in various studies of hybrid prediction with the GCA–SCA model assuming that the fixed effects in the mixed linear model are known (e.g. Seye et al. 2020). For an extension to the general case when fixed effects are unknown, we present formulas for calculating GBLUPs and $r_{a}$ for hybrids, GCA and SCA effects using well-known results from mixed models (Henderson 1975). In the theory part, our goal was to derive formulas connecting $r_{a}$ of hybrids to $r_{a}$ of their GCA and SCA effects and provide estimates for $r_{a}$ of hybrids, GCA and SCA effects. In the simulation part, our objective was to investigate $r_{a}$ and two types of estimates ($\hat{r}_{a}$ and $\tilde{r}_{a}$) for different types of hybrids (H0, H1, H2) and GCA of I0 and I1 lines under various scenarios differing in the relative importance of SCA effects and heritabilities. In particular, we examined how these statistics are influenced by the number of hybrids in the TS $\left( {N_{TS} } \right)$ and its composition regarding the number of parent lines $\left( {n_{TS} } \right)$ sampled from each parent population versus the number of crosses per parent line $\left( c \right)$ in the TS. Finally, we discuss the implications of our results for the optimized design of the TS in hybrid breeding programs.

## Theory

We begin by formulating the statistical model and providing formulas for calculating GBLUPs (= best linear unbiased predictors based on genomic data) and their variances for hybrid performance and GCA and SCA effects under a general mixed model. Let $F$ be the set of $n_{F}$ female lines and $M$ the set of $n_{M}$ male lines for which genomic data are available, and $H = F \times M$ the set of all $n_{F} \times n_{M}$ possible hybrid combinations in the factorial between the lines of $F$ and $M$. The $N_{TS}$ hybrids in the TS are a subset of $H$. The lines in sets $F_{1} \subset F$ and $M_{1} \subset M$, denoted as I1 lines, serve as female or male parents of at least one hybrid in the TS. (In our notation, we use capital and lower case letters for numbers referring to hybrids and parent lines, respectively). By contrast, the lines in $F_{0} = F\backslash F_{1}$ and $M_{0} = M\backslash M_{1}$ are referred to as I0 lines, because they are not used as parent of any hybrid in the TS.

We assume a fixed number $N_{{{\text{TS}}}}$ of TS hybrids, which depends primarily on the budget of the breeder assigned for phenotyping of hybrids. The goal is to choose a subset $HT \subset H$ as TS such that the prediction accuracy for untested hybrids $H\backslash HT$ is maximized. However, in hybrid breeding we have to consider that the PS consists of different subsets of hybrids $\Phi_{s, t}$ $\left( {s, t = 0, 1} \right)$, with $\Phi_{0, 0} = \left[ {F_{0} \times M_{0} } \right]$ comprising the H0 hybrids, $\Phi_{1, 0} = \left[ {F_{1} \times M_{0} } \right]$ and $\Phi_{0,1} = \left[ {F_{0} \times M_{1} } \right]$ comprising the H1 hybrids, and $\Phi_{1, 1} = \left[ {F_{1} \times M_{1} } \right]\backslash HT$ comprising the H2 hybrids. Owing to different relatedness to other members of the TS, the H0, H1, and H2 types of hybrids have different prediction accuracies (Technow et al. 2014).

The common model to subdivide the genotypic value $G_{i \times j}$ of a hybrid $i \times j$ from the cross of female line $i \in F$ with male line $j \in M$ is (Hallauer et al. 2010)

1 $$G_{i \times j} = \mu + g_{F,i} + g_{M,j} + s_{i \times j} ,$$

where $\mu$ is the mean of the hybrids in $H$, $g_{F,i}$ and $g_{M,j}$ are the GCA effects of $i$ and $j$, respectively, and $s_{i \times j}$ is the SCA effect of their hybrid combination. The mixed model linking the phenotypic data of the hybrids in the TS to the GCA of the female and male lines and the SCA of all possible hybrid combinations in $H$ can be written as

2 $${\varvec{y}}_{TS} = \varvec{X}\varvec{\beta } + {\varvec{Zu}} + {\varvec{e}},$$

with $$E\left( {{\varvec{y}}_{{{\text{TS}}}} } \right) = {\varvec{X}}\beta$$, $$E\left( {\varvec{u}} \right) = 0$$, $$E\left( {\varvec{e}} \right) = 0$$, $${\text{var}} \left[ {\begin{array}{*{20}c} {\varvec{u}} \\ {\varvec{e}} \\ \end{array} } \right] = \left[ {\begin{array}{*{20}c} {\varvec{G}} & 0 \\ 0 & {\varvec{R}} \\ \end{array} } \right]$$, $${\text{var}} \left[ {{\varvec{y}}_{TS} } \right] = {\varvec{V}} = {\varvec{R}} + {\varvec{ZGZ}}^{{\varvec{T}}}$$, where

3 $${\varvec{Z}} = \left[ {\begin{array}{*{20}c} {{\varvec{Z}}_{F} } & {{\varvec{Z}}_{M} } & {{\varvec{Z}}_{H} } \\ \end{array} } \right],\,{\varvec{u}} = \left[ {\begin{array}{*{20}c} {{\varvec{g}}_{F} } \\ {{\varvec{g}}_{M} } \\ {{\varvec{s}}_{H} } \\ \end{array} } \right]\quad {\text{and}} \quad\quad {\text{var}} \left[ {\begin{array}{*{20}c} {{\varvec{g}}_{F} } \\ {{\varvec{g}}_{M} } \\ {{\varvec{s}}_{H} } \\ \end{array} } \right] = {\varvec{G}} = \left[ {\begin{array}{*{20}c} {{\varvec{G}}_{F} } & 0 & 0 \\ 0 & {{\varvec{G}}_{M} } & 0 \\ 0 & 0 & {{\varvec{G}}_{H} } \\ \end{array} } \right]$$

Here, ${\varvec{y}}_{TS} = \left( {y_{k} } \right)_{{k \in {\text{TS}}}}$ is a vector of dimension $N_{{{\text{TS}}}}$ of phenotypic observations of the hybrids in the TS, ${\varvec{\beta}}$ is the vector of non-genetic fixed effects of the hybrids in the TS and ${\varvec{X}}$ the design matrix linking these effects to the observations in ${\varvec{y}}_{{{\text{TS}}}}$, ${\varvec{u}}$ is a vector of random effects with dimension $N = n_{F} + n_{M} + n_{F} \times n_{M}$, composed of the vectors ${\varvec{g}}_{F}$ and ${\varvec{g}}_{M}$ of GCA effects of all $n_{F}$ female and $n_{M}$ male lines in $F$ and $M$, respectively, and the vector ${\varvec{s}}_{H}$ of SCA effects of all $N_{H} = n_{F} \times n_{M}$ hybrid combinations in $H$, and ${\varvec{e}}$ is the residual error. The vectors ${\varvec{g}}_{F}$, ${\varvec{g}}_{M}$ and ${\varvec{s}}_{H}$ are assumed to be (i) pairwise uncorrelated because the parent lines are sampled independently from the female and male population, and (ii) arranged in the order of the numbering of the lines in set $F$ and $M$, respectively, and the element $s_{k}$ in vector ${\varvec{s}}_{H} = \left( {s_{k} } \right)$ with $k = \left( {i - 1} \right) \times n_{M} + j$ refers to $s_{i \times j}$ ${\varvec{Z}}_{F} ,$ ${\varvec{Z}}_{M}$ and ${\varvec{Z}}_{H}$ are incidence matrices relating the phenotypic data in ${\varvec{y}}_{TS}$ with the vectors ${\varvec{g}}_{F}$, ${\varvec{g}}_{M}$ and ${\varvec{s}}_{H}$, which have columns of zeros, if the respective line is $\in F_{0}$ or $\in M_{0}$ or the hybrid combination is $\in H\backslash HT$, respectively. ${\varvec{G}}_{F} = \sigma_{{{\text{gca}}F}}^{2} {\varvec{K}}_{F}$, ${\varvec{G}}_{M} = \sigma_{{{\text{gca}}M}}^{2} {\varvec{K}}_{M}$ and ${\varvec{G}}_{H} = \sigma_{{{\text{sca}}}}^{2} {\varvec{K}}_{H}$, where ${\varvec{K}}_{F}$ and ${\varvec{K}}_{M}$ are the kinship matrices among the $n_{F}$ female lines and among the $n_{M}$ male lines, respectively. The relationship matrix ${\varvec{K}}_{H}$ for SCA effects is obtained as Kronecker product ${\varvec{K}}_{H} = {\varvec{K}}_{F} \otimes {\varvec{K}}_{M}$, as originally shown for pedigree-based coancestries (Cockerham 1961). $\sigma_{{{\text{gca}}F}}^{2}$ and $\sigma_{{{\text{gca}}M}}^{2}$ are the GCA variances among unrelated homozygous lines from the female and male parent population, respectively, and $\sigma_{{{\text{sca}}}}^{2}$ the SCA variance of unrelated single cross hybrids between lines of the two parent populations, from which the lines in $F$ and $M$ were sampled. ${\varvec{R}}$ denotes the corresponding “error” matrix pertaining to the model in Eq. (2), where ${\varvec{R}} = \sigma_{e}^{2} {\varvec{I}}_{{N_{{{\text{TS}}}} }}$ is assumed in most cases. The elements of ${\varvec{K}}_{F}$ and ${\varvec{K}}_{M}$ can be calculated from genomic data using established methods (VanRaden 2008).

Using results of Henderson (1975), the best linear unbiased predictor (BLUP) $\hat{\varvec{u}}$ for vector ${\varvec{u}}$ is obtained as:

4 $$\hat{\varvec{u}} = {\varvec{GZ}}^{{\varvec{T}}} {\varvec{V}}^{ - 1} \left( {{\varvec{y}}_{{{\text{TS}}}} - \varvec{X\hat{\beta }}} \right) = {\varvec{GZ}}^{{\varvec{T}}} {\varvec{Py}}_{{{\text{TS}}}} = {\varvec{B}} {\varvec{y}}_{{{\text{TS}}}}$$

with $$\overset{\lower0.5em\hbox{$\smash{\scriptscriptstyle\frown}$}}{\beta } = \left( {{\varvec{X}}^{T} {\varvec{V}}^{ - 1} {\varvec{X}}} \right)^{ - 1} {\varvec{X}}^{T} {\varvec{V}}^{ - 1} {\varvec{y}}_{{{\text{TS}}}}$$, $${\varvec{P}} = {\varvec{V}}^{ - 1} - {\varvec{V}}^{ - 1} {\varvec{X}}\left( {{\varvec{X}}^{T} {\varvec{V}}^{ - 1} {\varvec{X}}} \right)^{ - 1} {\varvec{X}}^{T} {\varvec{V}}^{ - 1}$$,and $${\varvec{B}} = {\varvec{GZ}}^{{\varvec{T}}} {\varvec{P}} = \left[ {\begin{array}{*{20}c} {{\varvec{G}}_{F} {\varvec{Z}}_{F}^{T} {\varvec{P}}} \\ {{\varvec{G}}_{M} {\varvec{Z}}_{M}^{T} {\varvec{P}}} \\ {{\varvec{G}}_{H} {\varvec{Z}}_{H}^{T} {\varvec{P}}} \\ \end{array} } \right] = \left[ {\begin{array}{*{20}c} {{\varvec{B}}_{F} } \\ {{\varvec{B}}_{M} } \\ {{\varvec{B}}_{H} } \\ \end{array} } \right]$$ , yielding for the vectors GCA and SCA effects the BLUPs

5 $$\hat{\varvec{g}}_{F} = {\varvec{B}}_{F} {\varvec{y}}_{{{\text{TS}}}} ,\,\hat{\varvec{g}}_{M} = {\varvec{B}}_{M} {\varvec{y}}_{{{\text{TS}}}} ,\,\hat{\varvec{s}}_{H} = {\varvec{B}}_{H} {\varvec{y}}_{{{\text{TS}}}} .$$

Thus, we get for the variance of the BLUPs

6 $${\text{var}} \left( {\hat{\varvec{u}}} \right) = {\varvec{BVB}}^{T} = {\varvec{L}}$$

and

7 $${\text{var}} \left( {\hat{\varvec{g}}_{F} } \right) = {\varvec{B}}_{F} {\varvec{VB}}_{F}^{T} = {\varvec{L}}_{F} ,\,{\text{var}} \left( {\hat{\varvec{g}}_{M} } \right) = {\varvec{B}}_{M} {\varvec{VB}}_{M}^{T} = {\varvec{L}}_{M} \quad {\text{and}}\quad {\text{var}} \left( {\hat{\varvec{s}}_{FH} } \right) = {\varvec{B}}_{H} {\varvec{VB}}_{H}^{T} = {\varvec{L}}_{H}$$

Since all hybrids have a common mean $\mu$, selection among them can be based on prediction of $h_{i \times j} = g_{F,i} + g_{M,j} + s_{i \times j}$ and does not require prediction of $G_{k \times l}$. This corresponds to predicting ${\varvec{h}} = {\varvec{g}}_{F} \otimes 1_{{N_{M} }} + 1_{{N_{F} }} \otimes {\varvec{g}}_{M} + {\varvec{s}}_{H}$, or

8 $${\varvec{h}} = {\varvec{Wu}}\quad {\text{with}}\quad {\varvec{W}} = \left[ {\begin{array}{*{20}c} {{\varvec{I}}_{{n_{F} }} \otimes 1_{{n_{M} }} } & {1_{{n_{F} }} \otimes {\varvec{I}}_{{n_{M} }} } & {{\varvec{I}}_{{n_{F} \times n_{M} }} } \\ \end{array} } \right],$$

where ${\varvec{I}}_{n}$ refers to a unity diagonal matrix, $1_{n}$ to a unity vector of dimension $n$, and $\otimes$ denotes the Kronecker product. Because the BLUP of a linear function of random effects is equal to the linear function of the BLUPs of the random effects (Henderson 1984), the BLUP of ${\varvec{h}}$ and its variance are obtained as

9 $$\hat{\varvec{h}} = {\varvec{W}}\hat{{\varvec{u}}} = {\varvec{WBy}}_{TS} = \hat{\varvec{g}}_{F} \otimes 1_{{n_{M} }} + 1_{{n_{F} }} \otimes \hat{\varvec{g}}_{M} + \hat{\varvec{s}}_{H} = \left( {{\varvec{B}}_{F} \otimes 1_{{n_{M} }} + 1_{{n_{F} }} \otimes {\varvec{B}}_{M} + {\varvec{B}}_{H} } \right){\varvec{y}}_{{{\text{TS}}}}$$

and

10 $${\text{var}} \left( {\hat{\varvec{h}}} \right) = {\varvec{WBVB}}^{T} {\varvec{W}}^{T} = {\varvec{WLW}}^{T} = {\varvec{B}}_{F} {\varvec{VB}}_{F}^{T} \otimes {\varvec{J}}_{{n_{M} }} \quad\quad+ {\varvec{J}}_{{n_{F} }} \otimes {\varvec{B}}_{M} {\varvec{VB}}_{M}^{T} + {\varvec{B}}_{H} {\varvec{VB}}_{H}^{T} + 2 \quad\quad \times \left[ {1_{{n_{F} }}^{T} \otimes {\varvec{B}}_{F} {\varvec{VB}}_{M}^{T} \otimes 1_{{n_{M} }} + {\varvec{B}}_{F} {\varvec{VB}}_{H}^{T} \otimes 1_{{n_{M} }} + 1_{{n_{F} }} \otimes {\varvec{B}}_{M} {\varvec{VB}}_{H}^{T} } \right],$$

where ${\varvec{J}}_{n}$ is a $n \times n$ matrix of ones.

For any subset $\Phi \subset \left\{ {1,...,n_{F} + n_{M} + n_{F} \times n_{M} } \right\}$ of elements in ${\varvec{u}}$, we can express the prediction accuracy $r_{a}$ as correlation $r\left( {\hat{\varvec{u}}_{\Phi } ,{\varvec{u}}_{\Phi } } \right)$ between the true genetic values (TGV) ${\varvec{u}}_{\Phi }$ and their GBLUPs $\hat{\varvec{u}}_{\Phi }$:

11 $$r_{a} \left( {\hat{\varvec{u}}_{\Phi } } \right) = r\left( {\hat{\varvec{u}}_{\Phi } ,{\varvec{u}}_{\Phi } } \right) = \frac{{\mathop \sum \nolimits_{k \in \Phi } \left( {\hat{u}_{k} - \overline{\hat{u}}_{k} } \right)\left( {u_{k} - \overline{u}_{k} } \right)}}{{\sqrt {\left( {\mathop \sum \nolimits_{k \in \Phi k} \left( {\hat{u}_{k} - \overline{\hat{u}}_{k} } \right)^{2} } \right)\left( {\mathop \sum \nolimits_{k \in \Phi } \left( {u_{k} - \overline{u}_{k} } \right)^{2} } \right)} }} = \frac{{\hat{\varvec{u}}^{T} {\varvec{S}}_{\Phi } {\varvec{u}}}}{{\sqrt {\left( {\hat{\varvec{u}}^{T} {\varvec{S}}_{\Phi } \hat{\varvec{u}}} \right)\left( {{\varvec{u}}^{T} {\varvec{S}}_{\Phi } {\varvec{u}}} \right)} }},$$

where a bar denotes the mean of $\hat{u}_{k}$ or $u_{k}$ over $k \in \Phi$, ${\varvec{S}}_{\Phi } = {\varvec{I}}_{\Phi } - \frac{1}{\left| \Phi \right|}{\varvec{J}}_{\Phi }$ is a centering matrix such that ${\varvec{I}}_{\Phi }$ is a matrix of dimension $n_{F} + n_{M} + n_{F} \times n_{M}$ having values of 1 on the diagonal, if the corresponding index $n \in \Phi$, and zeros elsewhere, ${\varvec{J}}_{\Phi }$ is a matrix of the same dimension having 1’s, if both indices $k,l \in \Phi$, and zeros elsewhere, and $\left| \Phi \right|$ is the number of elements in $\Phi$. For hybrid values, we have for $\Phi \subset \left\{ {1,...,N_{H} = n_{F} \times n_{M} } \right\}$

12 $$r_{a} \left( {\hat{\varvec{h}}_{\Phi } } \right) = r\left( {\hat{\varvec{h}}_{\Phi } ,{\varvec{h}}_{\Phi } } \right) = \frac{{\hat{\varvec{u}}^{T} {\varvec{W}}^{T} {\varvec{S}}_{\Phi } {\varvec{Wu}}}}{{\sqrt {\left( {\hat{\varvec{u}}^{T} {\varvec{W}}^{T} {\varvec{S}}_{\Phi } \varvec{W\hat{u}}} \right)\left( {{\varvec{u}}^{T} {\varvec{W}}^{T} {\varvec{S}}_{\Phi } {\varvec{Wu}}} \right)} }}$$

In simulations with known values of ${\varvec{u}}$, this formula can be used to calculate $r_{a}$ for hybrid prediction. If the TGV are unknown, as is the case in practice, one could use cross-validation to determine the predictive ability $R_{a}$ of GBLUPs replacing in Eqs. (11 and 12) the TGV by phenotypic data and get an estimate of $r_{a}$ by the ratio $R_{a} /\sqrt {h^{2} }$, where $h^{2}$ is the heritability of the phenotypic data (cf. Dekkers 2007). However, cross-validation can be circumvented by an alternative approach suggested by Ould Estaghvirou et al. (2013). Accordingly, an approximation of $E\left[ {r\left( {\hat{\varvec{u}}_{\Phi } ,{\varvec{u}}_{\Phi } } \right)} \right]$ and consequently an estimate of $r_{a} \left( {\hat{\varvec{u}}_{\Phi } } \right)$ can be obtained for any subset $\Phi$ as (see Appendix 1):

13 $$\hat{r}_{a} \left( {\hat{\varvec{u}}_{\Phi } } \right) = \sqrt {\frac{{tr\left( {{\varvec{S}}_{\Phi } {\varvec{L}}} \right)}}{{tr\left( {{\varvec{S}}_{\Phi } {\varvec{G}}} \right)}}} = \sqrt {\frac{{\mathop \sum \nolimits_{i \in \Phi } \left( {l_{ii} - l_{i.} } \right)}}{{\mathop \sum \nolimits_{i \in \Phi } \left( {g_{ii} - g_{i.} } \right)}}}$$

and

14 $$\hat{r}_{a} \left( {\hat{\varvec{h}}_{\Phi } } \right) = \sqrt {\frac{{tr\left( {{\varvec{S}}_{\Phi } {\varvec{WLW}}^{T} } \right)}}{{tr\left( {{\varvec{S}}_{\Phi } {\varvec{WGW}}^{T} } \right)}}}$$

The matrices ${\varvec{G}}$ and ${\varvec{L}}$ can be calculated (*cf.* Equations (3 and 6)) without phenotypic data of hybrids using genomic data of the parent lines, variance components $\sigma_{gcaF}^{2}$, $\sigma_{gcaM}^{2}$, $\sigma_{sca}^{2}$, ${\sigma }_{e}^{2}$ estimated from previous breeding cycles and the incidence matrices ${\varvec{X}}$ (usually ${\varvec{X}}=1$) and ${\varvec{Z}}$ pertaining to the fixed and random effects in Eq. (2), respectively, and matrix ${\varvec{W}}$ as defined in Eq. (8).

As shown in Appendix 2, the expected individual prediction accuracy (${\rho }_{a}$) for a randomly chosen hybrid $i\times j$ can be approximated by ${\rho }_{a}$ of its SCA and parental GCA effects as

15 $$\rho_{a} \left( {\hat{h}_{i \times j} ,h_{i \times j} } \right) \approx \sqrt {\rho_{a}^{2} \left( {\hat{g}_{F,i} ,g_{F,i} } \right)\tau_{{{\text{gca}}F}} + \rho_{a}^{2} \left( {\hat{g}_{M,j} ,g_{M,j} } \right)\tau_{{{\text{gca}}M}} + \rho_{a}^{2} \left( {\hat{s}_{i \times j} ,s_{i \times j} } \right)\tau_{{{\text{sca}}}} } ,$$

where ${\tau }_{gcaF}=\frac{{\sigma }_{\mathrm{gca}F}^{2}}{{\sigma }_{G}^{2}}$, ${\tau }_{\mathrm{gca}M}=\frac{{\sigma }_{\mathrm{gca}M}^{2}}{{\sigma }_{G}^{2}}$ and ${\tau }_{\mathrm{sca}}=\frac{{\sigma }_{\mathrm{sca}}^{2}}{{\sigma }_{G}^{2}}$ is the proportion of the total genetic variance ${\sigma }_{G}^{2}$ = ${\sigma }_{\mathrm{gca}F}^{2}$ +${\sigma }_{\mathrm{gca}M}^{2}$ + ${\sigma }_{\mathrm{sca}}^{2}$ among unrelated hybrids attributable to the GCA and SCA variances, respectively, with ${\tau }_{\mathrm{gca}F}+{\tau }_{\mathrm{gca}M}+{\tau }_{\mathrm{sca}}=1.$ If set $\Phi$ is equal to ${\Phi }_{s,t}$ ($s,t=\mathrm{0,1}$), corresponding to the set of hybrids of type H0, H1 and H2, respectively, a similar approximation applies to the prediction accuracies ${r}_{a}$ of hybrids and GCA and SCA effects (see Appendix 2, Eq. (28)). This relationship can be used to obtain a further estimate $\tilde{r}_{a} \left( {\hat{\varvec{h}}_{{\Phi_{s,t} }} } \right)$ of $r_{a} \left( {\hat{\varvec{h}}_{{\Phi_{s,t} }} } \right)$ (see Appendix 2, Eq. (30))

16 $$\tilde{r}_{a} \left( {\hat{\varvec{h}}_{{\Phi_{s,t} }} } \right) = \sqrt {r_{a}^{2} \left( {\hat{\varvec{g}}_{{F,F_{s} }} } \right)\tau_{{{\text{gca}}F}} + r_{a}^{2} \left( {\hat{\varvec{g}}_{{M,M_{t} }} } \right)\tau_{{{\text{gca}}M}} + r_{a}^{2} \left( {\hat{\varvec{s}}_{{\Phi_{s,t} }} } \right)\tau_{{{\text{sca}}}} }$$

where $r_{a} \left( {\hat{\varvec{g}}_{{F,F_{s} }} } \right)$, $\tau_{{{\text{gca}}F}}$ etc. must be substituted by appropriate estimates. This relationship holds true for H0 and H1 hybrids irrespective of the structure of the TS. For H2 hybrids, the TS must have the structure of a balanced incomplete factorial design, where $\left|{F}_{1}\right|=\left|{M}_{1}\right|$ and each line $i\in {F}_{1}$ was crossed to the same number $c$ of parents from ${M}_{1}$ and vice versa.

### Genetic materials, markers and genomic relationships

We based our simulations on the SNP marker genotypes of maize inbreds from two experiments conducted by the maize breeding program of the University of Hohenheim. Since the lines were expected to be fully homozygous, heterozygous marker genotypes were treated as “missing.” Markers with more than 5% missing values were removed; otherwise, “missing” values were imputed using BEAGLE version 5.0 (Browning and Browning 2016). To avoid clustering of markers in small genomic segments, we restricted the number of markers to a maximum of 10 per Mbp and retained from markers with linkage disequilibrium *r*^*2*^ ≥ 0.999 only one. The marker genotypes of hybrids were inferred from the genotypes of their parent lines.

Data set DS1 comprised SNP data of ${n}_{F}=145$ dent lines (= females) and ${n}_{M}=111$ flint lines (= males) genotyped with the 50 k Illumina SNP chip MaizeSNP50 (Ganal et al. 2011). The lines had been evaluated for GCA of grain yield and other important agronomic traits in testcross trials and selected to be used as parents of hybrids evaluated in factorials analyzed in various studies on hybrid prediction with different types of “omics” data (Technow et al. 2014; Westhues et al. 2017; Schrag et al. 2018). From the original set of 26,795 polymorphic markers in the 256 lines, 13,813 markers remained after quality check and pruning, denoted as set $SNP1$. From these, 12,058 were polymorphic within the 145 dent lines and 12,053 within the 111 flint lines. None of the SNPs was polymorphic between the two groups but monomorphic within each group. The polymorphic markers provided a largely uniform coverage of the entire maize genome. The genetic diversity of the lines and their linkage disequilibrium (LD) structure has been described in detail elsewhere (Technow et al. 2012, 2014). Our analyses were based on the ${N}_{H}={n}_{F}\times {n}_{M}=\mathrm{16,095}$ hybrids that could be simulated from the crosses between the factorial of the female and male parent lines.

Data set DS2 included SNP data from an unpublished experiment with ${n}_{F}=182$ dent inbreds and ${n}_{M}=162$ flint inbreds developed by in vivo haploid induction (Chaikam et al. 2019) from a single cross of two elite dent founder parents and a single cross of two related (coancestry $f=1/4$) elite flint founder parents, respectively. The lines were unselected except for seed set in the first generation (*D*_0_) of doubled-haploid plants to secure efficient line multiplication. All lines and their four homozygous parents were genotyped with the 600 k Affymetrix^®^ Axiom^®^ Maize Array (Unterseer et al. 2014). The number of polymorphic markers in set $SNP2$ amounted to 107,135 SNPs, with 2428 remaining after pruning because many of them were completely linked, out of which 2157 SNPs segregated in the dent lines and 1311 in the flint lines, and 1040 in both populations. As expected for doubled-haploid lines from bi-parental crosses, there were numerous monomorphic regions in both crosses, especially for in the flint population as a consequence of the relatedness of the founder parents. All analyses were based on the ${N}_{H}={n}_{F}\times {n}_{M}=\mathrm{29,484}$ inter-population hybrids that could be produced in silico among the female and male parent lines.

For each data set, genomic kinship matrices ${{\varvec{K}}}_{F}$ and ${{\varvec{K}}}_{M}$ of the female and male lines were calculated with Method 1 of VanRaden (2008) using the respective parent population to determine the frequency of the reference allele. The matrix ${{\varvec{K}}}_{H}$ was subsequently obtained as the Kronecker product ${{\varvec{K}}}_{F}\otimes {{\varvec{K}}}_{M}$.

### Traits

To simulate traits by our software module A (Figure S1), we followed the procedure described in previous papers (Technow et al. 2012; Esfandyari et al. 2015; Seye et al. 2020) with modifications. Briefly, a random set $QP$ of 3000 SNPs from set $SNP1$ were chosen as possible QTL positions in DS1, with the restriction that the number of SNPs was approximately proportional to the genetic length of the chromosomes. In DS2 a random set $QP$ of 500 SNPs from set $SNP1$ were chosen as possible QTL with the restriction that number of QTL was proportional the map length of the polymorphic regions (DS2). A random subset $Q\subset QP$ of ${n}_{Q}$ QTL were assigned additive (${a}_{l}$) and dominance effects (${d}_{l}={a}_{l}\times {k}_{l}$), where ${k}_{l}$ is the degree of dominance, defined according to Lynch and Walsh (1998). The additive effects ${a}_{l}$ were drawn from a Gamma distribution with parameter scale = 1.66 and shape = 0.4 and assigned to the reference allele coded as 1 in the SNP data. The degree of dominance ${k}_{l}$ was drawn from a normal distribution $N\left({\mu }_{k}, {\sigma }_{k}^{2}\right)$. A subset ${Q}_{d}\subset Q$ of ${n}_{{Q}_{d}}$ QTL displaying only dominance effects ${d}_{l}$ was finally obtained by setting ${a}_{l}=0$. The parameters ${\mu }_{k},{\sigma }_{k}^{2}$ were chosen based on the average degree of dominance summarized by Hallauer et al. (2010) from numerous experiments with maize for grain yield, maturity, resistance and quality traits. QTL studies of heterotic traits in elite hybrids with the NC design III (Garcia et al. 2008; Schön et al. 2010), triple testcross design (Frascaroli et al. 2007), or the F2 and immortalized F2 design (Stuber et al. 1992; Tang et al. 2010; Guo et al. 2014) served as reference point for determining the ratio ${n}_{{Q}_{d}}{:n}_{Q}$. Table S1 shows the values of ${n}_{Q}$, ${n}_{{Q}_{d}}$, ${\mu }_{k}$, ${\sigma }_{k}$ leading to three types of “target” traits with ${\tau }_{sca}=1\%, 6\%, 22\%$, which in combination with $h^{2} = 0.4, 0.8$ define the six scenarios analyzed in our study. The plausibility of our model assumptions for simulating different types of traits was confirmed by the close agreement of the ${\tau }_{sca}$ values in the simulated hybrid populations with experimental estimates for yield, maturity, and quality traits from the maize literature (Table S2).

Based on the simulated QTL genotypes for set $Q$, the genotypic value of every hybrid was determined by summing the corresponding additive and dominance effects, respectively, across all QTL. Subsequently, the genotypic values were scaled to unit variance and centered to zero mean. Phenotypic values of the hybrids were obtained by adding to the genotypic values a normally distributed noise variable with variance ${\sigma }_{e}^{2}=1/{h}^{2}-1$ to obtain the desired broad sense heritabilities. By averaging the genotypic values over all hybrid combinations of a given line, we obtained its “true” GCA. The “true” SCA of each hybrid combination was obtained by subtracting the GCA of both parents from the genotypic value of the hybrid. Variance components ${\sigma }_{gcaF}^{2}$, ${\sigma }_{gcaM}^{2}$, ${\sigma }_{sca}^{2}$, ${\sigma }_{G}^{2}$ and the ratio ${\tau }_{sca}={\sigma }_{sca}^{2}{:\sigma }_{G}^{2}$ were determined from the GCA and SCA values of the complete factorial (= set $H$) and used for calculating GBLUPs as described below. For each of the six scenarios, simulation of each type of target trait was replicated 50 times by sampling always anew QTL positions and effects and the noise variable.

### Prediction of hybrids

**Fig. 1:**
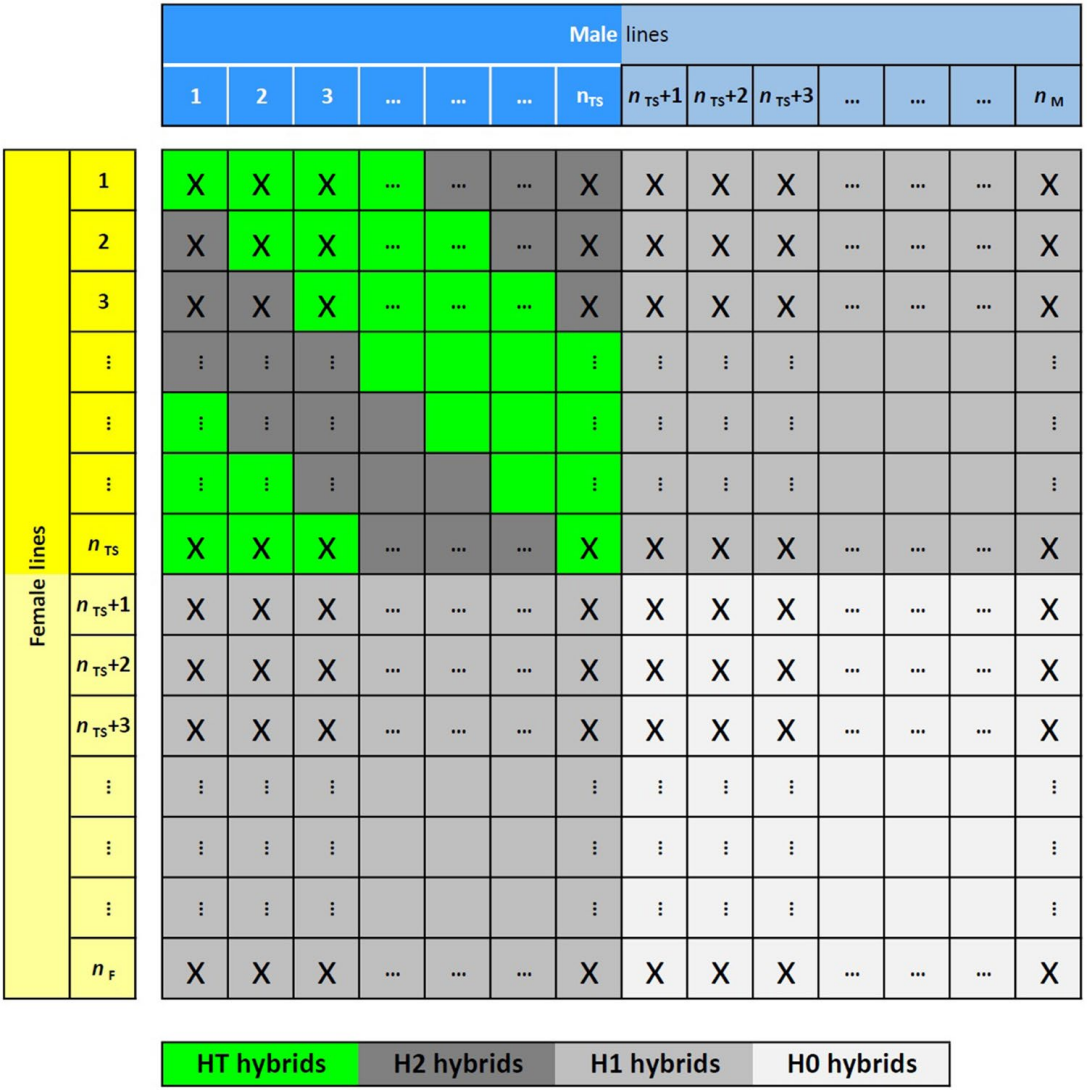
Schematic representation of the training set (TS) of hybrids (HT hybrids, ${N}_{TS}=28$ green) as determined by the number of lines (${n}_{TS}=7$) sampled from each parent population (females = yellow, males = blue) and crosses per parent line (here $c=4$) used for genomic prediction of hybrid performance and GCA of the parent lines. I0 and I1 lines are shown with weak and strong color intensity, respectively, and H0, H1 and H2 hybrids by increasing intensity levels of gray (color figure online)

For simulating the TS and PS for data set DS1 and DS2 in software module B (Figure S1), we first sampled randomly ${n}_{TS}$ lines from each of the sets $F$ and $M$ to obtain the subsets ${F}_{1}$ and ${M}_{1}$ of I1 lines for producing the TS hybrids. The parental lines were crossed such that each male from ${M}_{1}$ was mated to $c$ females from ${F}_{1}$ and each female from ${F}_{1}$ was mated to $c$ male lines from ${M}_{1}$ to obtain a total of ${N}_{\mathrm{TS}}={n}_{\mathrm{TS}}\times c$ hybrid combinations for the TS according to the scheme depicted in Fig. 1 for$c=4$. We varied ${n}_{\mathrm{TS}}=12, 24, \text{36, }..., 96$ and ${n}_{\mathrm{TS}}=12, 24, 36, ..., 144$ for DS1 and DS2, respectively, and $c=1, 2, 4$ to investigate the effect of the composition of the TS on the prediction accuracy for GCA of both I0 and I1 lines as well as the SCA and hybrid performance for H0*,* H1, and H2 hybrids marked by different colors in Fig. 1. Sampling of the subset of I1 lines in each parent population and production of crosses for generating the TS was repeated 20 times. Hence, for each scenario we had a total of 1000 simulation runs, corresponding to 50 replications for each type of target trait × 20 parent samplings for the TS.

**Fig. 2:**
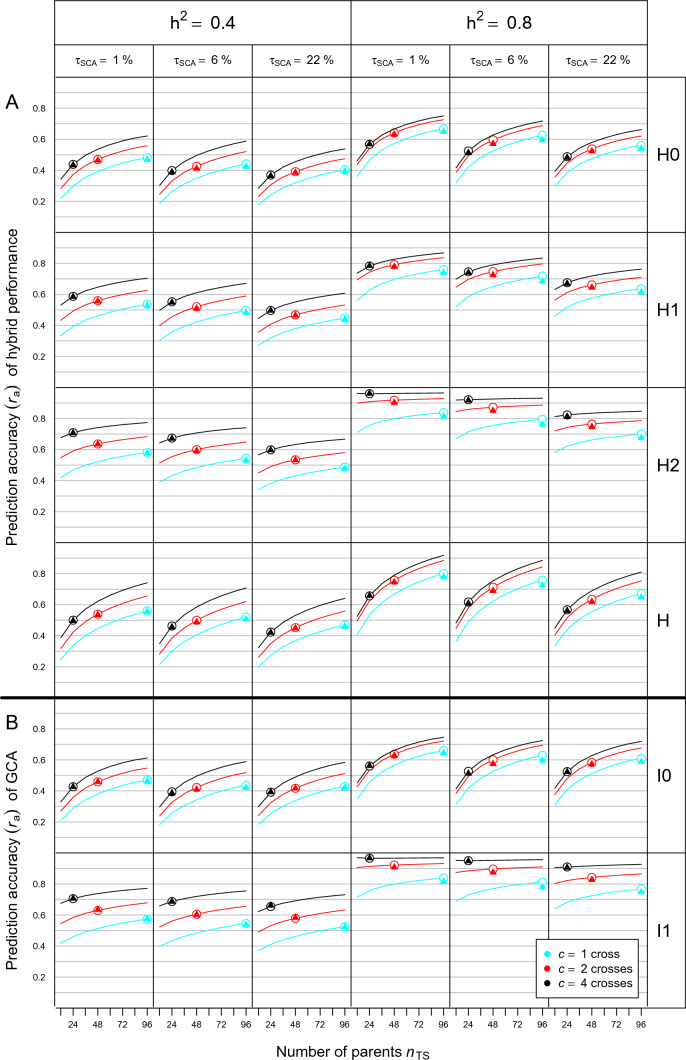
Prediction accuracy (${r}_{a}$) for **A** H0, H1, and H2 type of hybrids and all hybrids in set $H$ and **B** GCA of I0 and I1 lines as a function of the number ${n}_{TS}$ of parent lines and number of crosses per parent ($c=1, 2, 4$) used for producing the training set (TS). Results refer to means of 1000 simulation runs based on data set DS1 for different values of ${h}^{2}$ and ${\tau }_{SCA}$ (proportion of the SCA variance in ${\sigma }_{g}^{2}$ of hybrids). Circles and triangles refer to ${r}_{a}$ values for ${N}_{TS}={n}_{TS}\times c=96$ obtained with GBLUPs calculated with “true” and estimated variance components, respectively

**Fig. 3:**
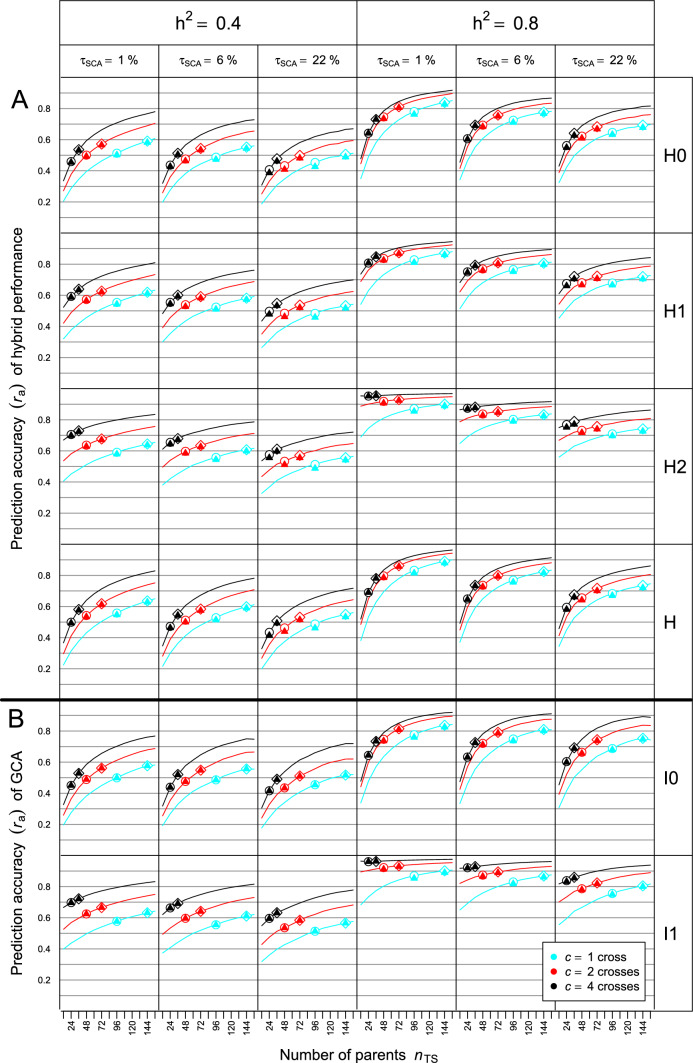
Prediction accuracy (${r}_{a}$) for **A** H0, H1, and H2 type of hybrids and all hybrids in set $H$ and **B** GCA of I0 and I1 lines as a function of the number ${n}_{TS}$ of parent lines and number of crosses per parent ($c=1, 2, 4$) used for producing the training set (TS). Results refer to means of 1000 simulation runs based on data set DS2 for different values of ${h}^{2}$ and ${\tau }_{SCA}$ (proportion of the SCA variance in ${\sigma }_{g}^{2}$ of hybrids). Circles and triangles refer to results for ${N}_{TS}={n}_{TS}\times c=96$ and diamonds and triangles refer to results for ${N}_{TS}={n}_{TS}\times c=144$ obtained with GBLUPs calculated with “true” and estimated variance components, respectively

In software module C (Figure S1), we performed GBLUP for genomic prediction of all hybrids in set $H$ and GCA effects of all lines in set $F$ and $M$ on the basis of the GCA–SCA model in Eq. (1). The variance components ${\sigma }_{\mathrm{gca}F}^{2}$, ${\sigma }_{\mathrm{gca}M}^{2}$, ${\sigma }_{\mathrm{sca}}^{2}$ calculated from set* H* as described above were used in our calculations, which enabled us to finish 1000 simulation runs for each scenario within acceptable time. This procedure is only feasible in simulations but in practice, estimated values of the variance components obtained from phenotypic values and genomic relationships in the TS must be employed in GBLUP. For comparison, we therefore calculated GBLUPs with variance components estimated from the phenotypic data and genomic relationships of the hybrids in the TS for ${N}_{\mathrm{TS}}=96$ in both data sets and ${N}_{\mathrm{TS}}=144$ in DS2. The variance components were estimated as posterior mean obtained by a Gibbs sampler with 2500 burn-ins and a chain length of 10,000 (Sorensen and Gianola 2002), which warranted satisfactory convergence. The close agreement of ${r}_{a}$ values obtained with both methods shown in Figs. 2 and 3 justified this procedure.

For final analysis, we calculated in each simulation run the prediction accuracy for genomic prediction of hybrids, GCA and SCA as Pearson correlation of their GBLUPs and their known genetic values across all genotypes in the respective set using Eqs. (11, 12). Furthermore, we calculated the approximation ${\widetilde{r}}_{a}$ of ${r}_{a}$ with Eq. (16). Likewise, ${\widehat{r}}_{a}$ of the GBLUPs was calculated by inserting the required population parameters (${{\varvec{K}}}_{F}$, ${{\varvec{K}}}_{M}$, ${{\varvec{K}}}_{H}$,${\sigma }_{\mathrm{gca}F}^{2}$, ${\sigma }_{\mathrm{gca}M}^{2}$, ${\sigma }_{\mathrm{sca}}^{2}$, ${\sigma }_{e}^{2}$) in Eqs. (13, 14). Finally, we calculated the mean of each statistic across the 1000 simulation runs for each scenario as well as the corresponding 95% confidence interval, which was for the ${r}_{a}$, ${\widehat{r}}_{a}$ and ${\widetilde{r}}_{a}$ values of all types of hybrids smaller than 1% of the mean. All computations were performed with the Julia programming language (Bezanson et al. 2017).

### Data availability statement

The marker data for sets DS1 and DS2, the positions of the markers and the Julia program are available at https://github.com/TUMplantbreeding/OptimTrainingSetDesign and can be downloaded from there.

## Results

Figure 2A shows the curves of ${r}_{a}$ for the three types (H0, H1, H2) of hybrids in the PS and all hybrids in set *H* of data set DS1 as a function of the number of parent lines ${n}_{\mathrm{TS}}$ and number of crosses $c=1, 2, 4$ per parent in the TS, which together determine the size of the TS (${N}_{\mathrm{TS}}={n}_{\mathrm{TS}}\times c$). For given ${h}^{2}$ and type of hybrid, the shape of the curves was almost congruent irrespective of $c$ and ${\tau }_{SCA}$, with a concave curvature that flattened out for larger ${n}_{TS}$ except for almost horizontal curves for H2 hybrids if ${h}^{2}=0.8$ and $c=4$. The level of ${r}_{a}$ increased substantially by doubling $c$ from 1 to 2 but a further doubling to $c=4$ yielded a much smaller increase for high ${h}^{2}$ as indicated by the distance between the curves. Increasing ${h}^{2}$ from 0.4 to 0.8 increased ${r}_{a}$ by ~ 30% for all types of hybrids. The ${r}_{a}$ curves differed little between ${\tau }_{\mathrm{SCA}}=1\%$ and 6% but were at a substantially lower level for ${\tau }_{\mathrm{SCA}}=22\%$. The ${r}_{a}$ values for all hybrids in $H$ followed closely the curves for H0 hybrids but with a steeper slope.

Using a fixed size ${N}_{\mathrm{TS}}=96$ of the TS as a benchmark (circles in Fig. 2A), the value of $c$ maximizing ${r}_{a}$ depended on the type of hybrid but for each type, the ranking of ${r}_{a}$ values for $c =1, 2, 4$ was identical for the six scenarios. For H0 hybrids, ${r}_{a}$ was maximum for $c=1$, exceeding ${r}_{a}$ values for $c=2$ and $c=4$ by ~ 1–3% and ~ 5–10%, respectively, with largest differences for high ${h}^{2}$. The ${r}_{a}$ values for H1 and H2 hybrids were always smallest for $c=1$ followed by $c=2$ and $c=4$, with differences being much larger for H2 than H1 hybrids. The curves of ${r}_{a}$ for GCA of I0 and I1 lines had a striking similarity with the corresponding curves for H0 and H2 hybrids, albeit at a slightly higher level (Fig. 2B).

The curves of ${r}_{a}$ for data set DS2 showed essentially the same picture as those for DS1, with some trends being amplified (Fig. 3). Again, the curves for H0 and H2 hybrids were almost identical with those for GCA of I0 and I1 lines, respectively. However, the curves had initially a steeper slope, especially for GCA of I0 lines, and approached a plateau at ${N}_{\mathrm{TS}}=144$ for high ${h}^{2}$. For ${N}_{\mathrm{TS}}=96$, the maximum ${r}_{a}$ value for H0 hybrids showed in all six scenarios a larger difference between $c=1$ and $c=4$ than for DS1. The maximum ${r}_{a}$ for $c=1$ referring to ${\tau }_{\mathrm{SCA}}=22\%$ and 1% ranged for low ${h}^{2}$ between 0.50 and 0.65 and for high ${h}^{2}$ between 0.70 and 0.85, respectively. By comparison, ${r}_{a}$ values of H1 hybrids hardly differed between $c=1, 2, 4$ for ${N}_{\mathrm{TS}}=96$ and ${N}_{\mathrm{TS}}=144$. For H2 hybrids and given values of ${N}_{TS}$, differences between ${r}_{a}$ for different values of $c$ were substantial for all scenarios, most notably for ${h}^{2}=0.4$. The prediction accuracy across all hybrids in set H had its maximum for $c=1$ in all scenarios. For ${N}_{\mathrm{TS}}=96$, the difference to ${r}_{a}$ for $c=2$ ranged between 1% (${h}^{2}=0.4$, ${\tau }_{\mathrm{sca}}=22\%$) and 14% (${h}^{2}=0.8$, ${\tau }_{\mathrm{sca}}=1\%$), but for ${N}_{TS}=144$, these differences became smaller.

For the subset of simulations with ${N}_{\mathrm{TS}}=96$ for DS1 and ${N}_{\mathrm{TS}}=96$ and 144 for DS2, the ${r}_{a}$ values for hybrid performance obtained with GBLUPs calculated with estimated variance components (triangles) were almost identical with the corresponding ${r}_{a}$ values of GBLUPs calculated with the “true” variance components (circles and diamonds) (Figs. 2 and 3). The latter estimates showed for ${h}^{2}=0.8$ a small upward bias of less than 3% for $c=1, 2$ in all types of hybrids. As expected the SD of ${r}_{a}$ values was generally much larger for ${r}_{a}$ values calculated with estimated variance components due to the estimation error associated with them (results not shown).

The ${r}_{a}$ values for SCA were for all types of hybrids in both data sets (Figures S2 and S3) much smaller than those for GCA of I0 and I1 lines. For all scenarios and types of hybrids, ${r}_{a}$ values were much lower for DS1 than DS2. For ${h}^{2}=0.4$, the prediction accuracy of SCA was lower than 0.28 for all cases. For ${h}^{2}=0.8$, the prediction accuracy was of moderate size for H2 hybrids in both data sets, but even in the most favorable case with ${\tau }_{\mathrm{SCA}}=22\%$ and $c=4$, ${r}_{a}$ for the hybrids in $H$ barely exceeded 0.4 for ${N}_{\mathrm{TS}}=144$ in DS2. Estimates of ${r}_{a}$ for SCA effects obtained from GBLUPs calculated with estimated variance components (triangles) were almost identical to those obtained with “true” variance components (circles and diamonds).

Calculating ${\widetilde{r}}_{a}$ by Eq. (16) as a function of the ${r}_{a}$ and $\tau$ values of GCA and SCA effects provided an excellent approximation of ${r}_{a}$ values for all scenarios and values of ${n}_{\mathrm{TS}}$ and $c$ in both data sets (Figures S4 and S5). Similarly, the curves for ${\widehat{r}}_{a}$ of hybrids and GCA effects calculated according to Eqs. (13, 14) had identical shape as the curves for ${r}_{a}$ in all scenarios (Figures S6 and S7). In both data sets, ${\widehat{r}}_{a}$ showed in comparison with ${r}_{a}$ a slight upward and minor downward bias for low and high ${h}^{2}$, respectively.

## Discussion

Our research pertains to the prediction of inter-population hybrids produced by crossing lines of two genetically distant populations. This setting is typical for established hybrid breeding programs in maize and other allogamous crops, because organizing the germplasm in genetically divergent parent populations warrants optimum exploitation of heterosis and reduces the proportion ${\tau }_{\mathrm{SCA}}$ of the SCA variance in ${\sigma }_{g}^{2}$ of hybrids according to experimental results (Melchinger and Gumber 1998) and theoretical arguments (Reif et al. 2007). Our simulations show that smaller values of ${\tau }_{\mathrm{SCA}}$ increase the prediction accuracy for all types of hybrids in the PS, irrespective of the data set and the size and design of the TS (Figs. 2 and 3). Thus, our results support the conclusion of Zhao et al. (2015) that genetically distant heterotic groups are advantageous for both conventional hybrid breeding and implementation of genomic prediction.

### Choice of trait architecture, parent populations and genetic model

By using simulations, we were able to investigate various scenarios in hybrid breeding with a large number of replications and to determine the prediction accuracy directly by correlating predicted and “true" genotypic values, thereby bypassing the estimation of ${r}_{a}$ by means of cross-validation. Following Fisher (1918), we assumed a large number of QTL underlying the genetic architecture of complex quantitative traits with small additive and dominance effects as practiced in previous studies with similar objectives (Technow et al. 2012; Seye et al. 2020). We ignored epistasis given its minor importance in experimental studies with maize (Melchinger et al. 1988; Lamkey et al. 1995) and the low importance of statistical epistasis at the level of populations even in the presence of significant physiological epistasis at the level of individual genotypes (Hill et al. 2008; Sackton and Hartl 2016).

The genotypic data underlying our simulations were taken from an active maize breeding program to warrant high practical relevance. Data set DS1 represents the situation when numerous preselected lines are available and a limited number of most promising hybrid combinations are to be produced and evaluated for final product development. The lines in DS1 had been selected based on their line perse and testcross performance but we expect similar outcomes if they were identified with genomic selection. Data set DS2 can be viewed as an application of the Hallauer (1967) proposal of full-sib selection for hybrid breeding, where single crosses instead of testcrosses are evaluated at each stage of the breeding cycle, in which case genomic prediction is the only way to exploit the effects of Mendelian sampling in the parent populations.

We limited our investigation to the classical GCA–SCA model of Sprague and Tatum (1942) for modeling hybrid performance. Kadam et al. (2021) used a model with only additive effects for investigating the optimum TS composition, whereas Fristche-Neto et al. (2018) used additionally a model with both additive and dominance effects. The GCA–SCA model has the advantage that it captures population-specific effects of SNPs, which is important if the linkage phase and strength of linkage disequilibrium (LD) between QTL and SNPs and/or the QTL effects differ between the parent populations. A systematic comparison of the prediction accuracy of the different models is missing in the literature and warrants further research with experimental data but we do not expect that choice of the model will strongly affect the optimum design of the TS.

We restricted our analyses to GBLUPs for four reasons. First, GBLUP allowed to calculate (i) an approximation ${\widehat{r}}_{a}$ of the expectation of the prediction accuracy ${r}_{a}$ for hybrid performance (Eq. (14)) and GCA and SCA effects (Eq. (13)) based on population parameters, and (ii) an approximation ${\widetilde{r}}_{a}$ of ${r}_{a}$ as function of ${r}_{a}$ estimates for GCA and SCA effects (Eq. (16)). Second, BLUP is relatively simple to compute and under multivariate normality, it is the conditional mean, which has well-known optimality properties for selection based on predicted values (Fernando and Gianola 1986). Third, GBLUP proved to be competitive in comparison with other parametric and nonparametric methods for prediction targeting a single population (Heslot et al. 2012; Crossa et al. 2013) or a hybrid population (Kadam and Lorenz 2019). Fourth, GBLUP can be easily adopted to cope with genotype × environment interactions (Ferrão et al. 2020) and epistasis by using appropriate Gaussian kernels based on ordinary genomic relationship matrices (Jiang and Reif 2015).

### Prediction accuracy of hybrids, GCA and SCA effects

By assuming absence of covariances between GBLUPs of GCA and SCA effects, we were able to derive ${\widetilde{r}}_{a}$ in Eq. (16) as an approximation of ${r}_{a}$ for hybrid performance, which depends on the ${r}_{a}$ of GCA and SCA effects and their contribution to ${\sigma }_{G}^{2}$. As confirmed by almost identical curves for ${\widetilde{r}}_{a}$ and ${r}_{a}$ (Figures S4 and S5), Eq. (16) yielded for both data sets an excellent approximation of the prediction accuracy of hybrids, which provides a key for their interpretation in the light of ${r}_{a}$ for GCA and SCA effects.

For all scenarios, we observed striking differences in the composition of the TS maximizing ${r}_{a}$ (Figs. 2 and 3). As known from other applications of genomic prediction in breeding (Clark et al. 2012; Riedelsheimer et al. 2013; Auinger et al. 2021), the degree of relationship between the genotypes in the TS and PS has a strong influence on the prediction accuracy. In the case of I1 lines, the cross(es) of each line in the TS can be regarded as member(s) of the virtual family of half-sibs underlying the definition of its GCA. Thus, increasing $c$ results for each I1 line in more hybrid relatives in the TS, which is expected to improve ${r}_{a}$ of its own GCA and that of I0 lines related to it. However, there will be fewer of such I1 lines that can contribute to ${r}_{a}$ of I0 lines. Therefore, one must be cautious in generalizing that $c=1$ is always the best choice as suggested by our findings.

Prediction of SCA was not promising for all scenarios in both data sets, because ${r}_{a}$ was generally too low for all sets of hybrids (Figure S2 and S5). Even in the most favorable case (${h}^{2}=0.8$, ${\tau }_{\mathrm{SCA}}=22\%$, H2 hybrids), a TS with ${n}_{\mathrm{TS}}=144$ and $c=4$ was needed in data set DS2 to achieve ${r}_{a}\sim 0.5$. However, this result may depend on the large genetic distance among the parent populations in our study and might differ, if no clearly defined heterotic groups are available as applies to autogamous crops at the beginning of hybrid breeding, where ${\tau }_{\mathrm{SCA}}$ can exceed 25%.

Combining the statements of the previous sections provides an explanation why the curves for prediction accuracy of H0 and H2 hybrids were almost identical to those for GCA of I0 and I1 lines, respectively, and the curves of H1 hybrids are in between those for H0 and H2 hybrids. Firstly, ${\widetilde{r}}_{a}$ is in close agreement with ${r}_{a}$ for all types of hybrids and all scenarios in both data sets (Figs. 2 and 3), indicating that Eq. (16) provides a solid basis for assessing the importance of GCA and SCA effects in hybrid prediction. Secondly, the contribution of SCA effects to ${\widetilde{r}}_{a}$ is close to zero, because ${\tau }_{SCA}$ and even more so ${r}_{a}^{2}$ for SCA effects are minor in comparison with the contribution of GCA effects. Thus, it follows that prediction accuracy of hybrids depends almost exclusively on ${r}_{a}$ of GCA effects.

The steeper increase in ${r}_{a}$ values for GCA of I0 lines and the higher level of the curves for data set DS2 than DS1 (Figs. 2 and 3) can be explained by differences in the structure of their parent populations. Data set DS1 included some related lines and displayed a rapid decay of LD between adjacent loci (cf. Technow et al. 2014). Consequently, pedigree relationships captured by markers were presumably an important driver of prediction accuracy. By comparison, because the DH lines of data set DS2 had undergone only one generation of genetic recombination, large haploblocks were present in each parent population as reflected by the large number of completely linked markers over long physical distances. Therefore, co-segregation of QTL and markers and high LD among them were most likely the main reasons for reaching very high levels of prediction accuracy even with moderate TS size (Habier et al. 2013; Schopp et al. 2017).

### Importance of additional factors influencing prediction accuracy

For a given number of field plots available for phenotyping, the breeder has in addition to the choice of $c$ the option to increase ${N}_{\mathrm{TS}}$ at the expense of evaluating the TS in fewer environments. However, in all scenarios doubling ${N}_{\mathrm{TS}}$ had generally a much smaller effect on increasing ${r}_{a}$ than doubling ${h}^{2}$, which increased prediction accuracy for all sets of hybrids by ~ 30% (Figs. 2 and 3). High ${h}^{2}$, which of course would need more than doubling the number of test environments, was particularly important for reliable prediction of H0 hybrids. Furthermore, increasing ${N}_{\mathrm{TS}}$ was more rewarding under high than low ${h}^{2}$, indicating that low ${h}^{2}$ can only partly be compensated by larger ${N}_{\mathrm{TS}}$.

Increasing ${n}_{TS}$ beyond 60 resulted in a rapidly diminishing increase in ${r}_{a}$ except for H0 hybrids and this held true for all values of $c$. For data set DS2, this can be explained by the large haplotypes in the parent populations similar to the results obtained with testcrosses of lines from bi-parental populations (Lehermeier et al. 2014; Lian et al. 2014). However, for data set DS1 this was unexpected and most likely attributable to its breeding history, because in genomic prediction with testcross data (Albrecht et al. 2014; Krchov and Bernardo 2015; Auinger et al. 2021), ${r}_{a}$ approached a plateau at much larger TS sizes. Consequently, regarding the optimum allocation of resources assigned to the TS and PS, one reaches soon the point, where a further increase in ${n}_{TS}$ and/or $c$ hardly pays off in a higher prediction accuracy.

Larger contributions (${\tau }_{\mathrm{SCA}}=22\% vs. 1\%$) of SCA to ${\sigma }_{G}^{2}$ of hybrids reduced ${r}_{a}$ for all scenarios and types of hybrids by less than 10% (Figs. 2 and 3). Hence, the decision on the optimum design of the TS is largely independent of the degree of heterosis in trait expression. This conclusion can be extended to the complexity of the trait as confirmed by simulations with smaller numbers of QTL (data not shown).

### Optimum design of the TS for genomic prediction of hybrids and GCA

Optimal implementation of GP in breeding programs requires a balanced compromise between the expenditures spent on the TS and PS (Riedelsheimer and Melchinger 2013) whereby the former influences mainly the prediction accuracy and the latter the selection intensity. Fortunately, the optimal design of the TS for prediction of hybrids and for prediction of GCA effects coincides due to almost perfect congruency of their curves for ${r}_{a}$ shown in Figs. 2 and 3. A further important finding was that the heritability and genetic trait architecture had essentially no influence, because for a given ${N}_{\mathrm{TS}}$ and type of hybrids, the ranking of ${r}_{a}$ values was identical, independent of ${h}^{2}$ and ${\tau }_{\mathrm{SCA}}$.

Nevertheless, the task of finding the optimal TS in hybrid breeding is complicated by the fact that one has to deal with three types (H0, H1, H2) of hybrids differing in their prediction accuracy. For $c=1$, the prediction accuracy was highest for H0 hybrids but lowest for H1 and H2 hybrids. Moreover, depending on the design of the TS, the composition of the PS will also change. If for example in DS1 and ${N}_{\mathrm{TS}}=96$, $c$ is increased from 1 to 4 and consequently ${n}_{\mathrm{TS}}$ is reduced by ¼, the number of H2 hybrids will be reduced by ~ 1/16 whereas the number of H0 hybrids will grow from 4.6 to 65.4%. Hence, in most cases H2 and HT hybrids have by far the lowest proportion in $H$ as the size of these sets depends on ${n}_{TS}$, which is generally small due the limited size of the TS as a result of the high costs of phenotyping. By comparison, H0 hybrids will represent by far the largest subset in $H$ as the cost of producing and genotyping a large number of lines in each parent population is rather inexpensive with the use of modern technologies such as doubled-haploid production and genotyping by sequencing, respectively. Thus, in most cases it will be most advantageous to have $c=1$ for designing the TS as confirmed by the prediction accuracy calculated across the entire set $H$ for ${N}_{\mathrm{TS}}=96$ in data set DS1 (Fig. 2) and ${N}_{\mathrm{TS}}=96\text{ and }144$ in DS2 (Fig. 3). This conclusion is in line with the results of the experimental study of Lorenzi et al. (2022) demonstrating the potential of sparse factorial designs for genomic prediction in hybrid breeding.

An exact solution of the optimization problem would require calculating the selection gain under truncation selection in multiple populations with different prediction accuracies and costs for H0, H1 and H2 hybrids, which is beyond the scope of this study. Obtaining reliable estimates of ${r}_{a}$ for each set of hybrids by cross-validation would require a large TS, exceeding by far the capacity of most breeding programs. As a practicable alternative, one might consider to determine ${\widehat{r}}_{a}$ for each type of hybrid, which can be calculated from genomic data and estimates of the relevant variance components that could be borrowed from previous breeding cycles. Using ${\widehat{r}}_{a}$ for optimizing the allocation of resources would also offer flexibility with regard to the choice of ${n}_{TS}$, $c$ and ${h}^{2}$ in order to balance the sample size ${N}_{\mathrm{TS}}$ versus the number of test environments used in phenotyping the TS for a given total number of test plots.

Besides genetic and economic aspects for the optimum design of the TS when adopting the “factorial” approach, breeders might prefer to use $c=1$ for practical reasons. First, fewer seeds are required from each parent, which may obviate the necessity of seed multiplication and thereby the loss of one generation, if seed multiplication is a problem, as applies often to the production of doubled haploids. Second, nicking in flowering of the female and male parents is more likely to be successful for a single pair than if two or more crosses are to be produced per parent in a partially balanced incomplete factorial design. If chemical agents or partitions are used for producing seed for testing purposes, only a corresponding spatial arrangement of the female and male genotypes is required.

In our simulations, the parents of the TS were randomly sampled from the set $F$ and $M$ of all females and males, respectively. In practice, however, the breeder has the option of a target sampling. Fristche-Neto et al. (2018) used the prediction error variance of the hybrids in the PS as selection criterion. They found a slightly higher prediction accuracy for selected over randomly chosen genotypes, when applying an additive model to factorial crosses, but this trend was reversed, when dominance effects were included. Kadam et al. (2021) also used an additive model in combination with the CDmean criterion of Rincent et al. (2012) applied to the hybrid population. With regard to applications of the GCA–SCA model, we suggest to perform the selection separately in each parent population, using search algorithms and criteria recently described for a single population (Isidro y Sánchez et al. 2022; Rio et al. 2022), but to replace the genetic variance in the formulas by the GCA variance of the respective parent population. The TS for hybrid prediction would then be produced by randomly crossing the lines selected from each parent population. In the presence of population structure, this method is expected to improve the prediction accuracy but further research is warranted to assess its merits.

### Topics for further research

In established hybrid breeding programs, the majority of activities deals with recycling breeding, where numerous (un-)related bi-parental families are produced anew in each parent population and cycle. As demonstrated with experimental data (Lehermeier et al. 2014) and simulations (Brauner et al. 2019), a genomic model trained with ~ 10 half-sib families of ~ 100 DH lines can yield the same prediction accuracy for testcross prediction of DH lines within a bi-parental family as if trained with ~ 100 full sibs from the same family. Hence, one might argue that including numerous related families of inter-population hybrids in the TS might also be possible for hybrid prediction. Seye et al. (2020) used this approach and obtained for all types of hybrids very high prediction accuracies that were calculated by pooling all 36 hybrid families. However, it remains unknown to what extent these results apply to genomic prediction of hybrids within families, which is the only way to exploit the within family variance and therefore of main interest to the breeder. When including members of all families in the PS, the variation among family means explains one third of the total additive genetic variance among the hybrids so that predicting their performance by their family mean would yield already a prediction accuracy of $\sqrt{1/3}=0.58$. A direct transfer of the results for genomic prediction across families for testcross performance to that for hybrid performance seems problematic because in the former case the gametic array of the tester is identical for all candidates, whereas in the latter case the gametes of the hybrids in the TS would mostly be sampled from different families. Thus, further research with well-designed experiments is warranted to examine the variation in the prediction accuracy of genomic prediction of hybrids between and across hybrid families similar to the study of Lehermeier et al. (2014) on testcross performance.

Our theoretical results were presented for homozygous lines but they also apply to heterozygous parents. Compared with homozygous lines, using heterozygous *S*_0_ plants as parents reduces the GCA variances by ½ and the SCA variance by ¼ so that ${\tau }_{sca}$ is expected to be very small even for highly heterotic traits. We therefore conjecture that the optimum design of the TS for this scenario will not fundamentally differ from that for homozygous parents. Confirmation of this hypothesis with simulations would be important for breeding of crops such as oil palm, where hybrids are produced from crosses between heterozygous parents and genomic selection holds great promise due to the drastic reduction of the generation interval (Cros et al. 2015; Kwong et al. 2017).

Further progress for genomic prediction is expected from machine learning methods (Yin et al. 2020) because they do not require assumptions about the distribution of marker effects or their statistical independence and are particularly suited to recognize and exploit patterns in the data regarding the action and interaction of alleles and similarities of genotypes. However, they need to be adapted to the special features of hybrid populations. Thus, these methods may further improve the already high prediction accuracy achieved by GBLUP, but again we do not expect that this will fundamentally change the optimum design of the TS in hybrid breeding unless the parent population have a strong population structure or other type of pattern.

## Conclusions

Genomic prediction holds great promise to cope with the huge number of potential hybrids enabled by recent progress in the development of new lines and high-throughput genotyping. Our simulations clearly show that the prediction accuracy of hybrids depends largely on the GCA of their parent lines. Therefore, optimizing the design of the TS for fixed ${N}_{\mathrm{TS}}$ goes hand in hand with the prediction of hybrids in product development and the prediction of GCA of the parent lines in recurrent selection.

We found that including one cross per parent line ($c=1$) in the TS yields highest prediction accuracy for H0 hybrids and GCA of I0 lines but lowest prediction accuracy for H2 hybrids and GCA of I1 lines, with H1 hybrids taking an intermediate position. The optimal design of the TS is therefore complicated by these opposite trends and depends heavily on the fraction of H0, H1 and H2 hybrids and I0 and I1 lines in the entire sets $H$*,* $F$ and $M$. A solution for this problem is calculating the expected selection response across all sets of hybrids and lines, taking into consideration the costs of genotyping versus phenotyping and other relevant aspects. However, if the bulk of predicted genotypes are H0 hybrids due to the low costs of genotyping, $c=1$ seems to be generally the best option for constructing the TS as confirmed by our results on the prediction accuracy across all types of hybrids in set $H$ in both data sets. With regard to the entailed paradigm shift in hybrid breeding from the “testcross” to the “factorial” approach, we recommend to complement our simulations with investigations based on experimental data and examine also the potential influence of epistasis and genotype × environment interactions on the optimum design of the TS.

### Electronic supplementary material

Below is the link to the electronic supplementary material.

Supplementary file1 (PDF 1218 KB)

## Appendix 1

### Derivation of the parametric estimate $\hat{r}_{a}$ of prediction accuracy for hybrids and their GCA and SCA effects

The expectation of the prediction accuracy $r_{a} \left( {\hat{\varvec{u}}_{\Phi } } \right)$ in Eq. (11) is

17 $$E\left[ {r\left( {\hat{\varvec{u}}_{\Phi } ,{\varvec{u}}_{\Phi } } \right)} \right] = E\left[ {\frac{{\hat{\varvec{u}}^{T} {\varvec{S}}_{\Phi } {\varvec{u}}}}{{\sqrt {\left( {\hat{\varvec{u}}^{T} {\varvec{S}}_{\Phi } \hat{\varvec{u}}} \right)\left( {{\varvec{u}}^{T} {\varvec{S}}_{\Phi } {\varvec{u}}} \right)} }}} \right]$$

Following Ould Estaghvirou et al. (2013), we approximate the expectation of the ratio and product of random variables by the ratio and product of their expectations

18 $$E\left[ {r\left( {\hat{\varvec{u}}_{\Phi } ,{\varvec{u}}_{\Phi } } \right)} \right] \approx \frac{{E\left[ {\hat{\varvec{u}}^{T} {\varvec{S}}_{\Phi } {\varvec{u}}} \right]}}{{\sqrt {E\left[ {\hat{\varvec{u}}^{T} {\varvec{S}}_{\Phi } \hat{\varvec{u}}} \right]E\left[ {\hat{\varvec{u}}^{T} {\varvec{S}}_{\Phi } \hat{\varvec{u}}} \right]} }}$$

Using results about (i) the expectation of bilinear forms (Searle 1971, p.65), and (ii) ${\text{cov}} \left( {\hat{\varvec{u}},{\varvec{u}}} \right) = {\text{var}} \left( {\hat{\varvec{u}}} \right)$ (Henderson 1975, p.425), we have

19 $$E\left[ {\hat{\varvec{u}}^{T} {\varvec{S}}_{\Phi } {\varvec{u}}} \right] = tr\left( {{\varvec{S}}_{\Phi } {\text{cov}} \left( {\hat{\varvec{u}},{\varvec{u}}} \right)} \right) = {\text{tr}}\left( {{\varvec{S}}_{\Phi } {\text{var}} \left( {\hat{\varvec{u}}} \right)} \right) = {\text{tr}}\left( {{\varvec{S}}_{\Phi } {\varvec{L}}} \right)$$

20 $$E\left[ {\hat{\varvec{u}}^{T} {\varvec{S}}_{\Phi } \hat{\varvec{u}}} \right] = {\text{tr}}\left( {{\varvec{S}}_{\Phi } {\text{cov}} \left( {\hat{\varvec{u}},\hat{\varvec{u}}} \right)} \right) = {\text{tr}}\left( {{\varvec{S}}_{\Phi } {\text{var}} \left( {\hat{\varvec{u}}} \right)} \right) = {\text{tr}}\left( {{\varvec{S}}_{\Phi } {\varvec{L}}} \right)$$

and

21 $$E\left[ {{\varvec{u}}^{T} {\varvec{S}}_{\Phi } {\varvec{u}}} \right] = {\text{tr}}\left( {{\varvec{S}}_{\Phi } {\text{cov}} \left( {{\varvec{u}},{\varvec{u}}} \right)} \right) = {\text{tr}}\left( {{\varvec{S}}_{\Phi } {\varvec{G}}} \right)$$

with ${\varvec{L}} = {\varvec{BVB}}^{T}$ (see Eq. (6)). Inserting these expressions in Eq. (18), we obtain $\hat{r}_{a} \left( {\hat{\varvec{u}}_{\Phi } } \right)$ as an estimate of $r_{a} \left( {\hat{\varvec{u}}_{\Phi } } \right)$

22 $$\hat{r}_{a} \left( {\hat{\varvec{u}}_{\Phi } } \right) = \sqrt {\frac{{{\text{tr}}\left( {{\varvec{S}}_{\Phi } {\varvec{L}}} \right)}}{{{\text{tr}}\left( {{\varvec{S}}_{\Phi } {\varvec{G}}} \right)}}} = \sqrt {\left\{ {\mathop \sum \limits_{i \in \Phi } \left( {l_{ii} - l_{i.} } \right)} \right\}/\mathop \sum \limits_{i \in \Phi } \left( {g_{ii} - g_{i.} } \right)}$$

where $${\varvec{G}} = \left( {g_{ij} } \right)$$, $${\varvec{L}} = \left( {l_{ij} } \right)$$, $$g_{{i.}} = \frac{1}{{\left| \Phi \right|}}\sum\nolimits_{{j \in \Phi }} {g_{{ij}} }$$ and $$l_{i.} = \frac{1}{\left| \Phi \right|}\mathop \sum \nolimits_{j \in \Phi } l_{ij}$$.

An estimate of $r_{a}$ for a subset $\Phi \subset \left\{ {1,...,N_{F} \times N_{M} } \right\}$ of hybrids is obtained as

23 $$\hat{r}_{a} \left( {\hat{\varvec{h}}_{\Phi } } \right) = \frac{{E\left( {\hat{\varvec{h}}^{T} {\varvec{S}}_{\Phi } {\varvec{h}}} \right)}}{{\sqrt {E\left( {\hat{\varvec{h}}^{T} {\varvec{S}}_{\Phi } \hat{\varvec{h}}} \right)E\left( {{\varvec{h}}^{T} {\varvec{S}}_{\Phi } {\varvec{h}}} \right)} }} = \sqrt {\frac{{{\text{tr}}\left( {{\varvec{S}}_{\Phi } {\varvec{WLW}}^{T} } \right)}}{{{\text{tr}}\left( {{\varvec{S}}_{\Phi } {\varvec{WGW}}^{T} } \right)}}}$$

Note that matrices ${\varvec{G}}$, ${\varvec{B}}$ and $\varvec{L }$ can be calculated from the genomic data of the genotypes in set $F$ and set $M$ (i.e., matrices ${\varvec{K}}_{F}$ and ${\varvec{K}}_{M}$), variance components $\sigma_{{{\text{gca}}F}}^{2}$, $\sigma_{{{\text{gca}}M}}^{2}$, $\sigma_{{{\text{sca}}}}^{2}$, $\sigma_{e}^{2}$, and the incidence matrices ${\varvec{X}}$ (usually ${\varvec{X}} = 1$) and ${\varvec{Z}}$ pertaining to the fixed and random effects in Eq. (2), respectively.

## Appendix 2

### Linking the prediction accuracies of hybrids to those of their SCA and parental GCA effects and derivation of $$\overset{\lower0.5em\hbox{$\smash{\scriptscriptstyle\smile}$}}{r}_{a}$$ for estimating the prediction accuracy of hybrids

Henderson (1975) proved for BLUP that ${\text{cov}} \left( {{\varvec{u}},\hat{\varvec{u}}} \right) = {\text{var}} \left( {\hat{\varvec{u}}} \right)$. Thus, the expected (individual) prediction accuracy of a randomly chosen hybrid $i \times j$ can be obtained by extracting the corresponding diagonal elements from the matrices ${\text{var}} \left( {\hat{\varvec{h}}} \right)$ and ${\text{var}} \left( {\varvec{h}} \right)$

24 $$\rho_{a} \left( {\hat{h}_{i \times j} ,h_{i \times j} } \right) = \frac{{{\text{cov}} \left( {\hat{h}_{i \times j} ,h_{i \times j} } \right)}}{{\sqrt {{\text{var}} \left( {\hat{h}_{i \times j} } \right){\text{var}} \left( {h_{i \times j} } \right)} }} = \sqrt {\frac{{{\text{var}} \left( {\hat{h}_{i \times j} } \right)}}{{{\text{var}} \left( {h_{i \times j} } \right)}}}$$

Ignoring covariances among BLUPs of GCA and SCA effects, we get from Eq. (10)

25 $${\text{var}} \left( {\hat{\varvec{h}}} \right) \approx {\text{var}} \left( {\hat{\varvec{g}}_{F} } \right) \otimes {\varvec{J}}_{{N_{M} }} + {\varvec{J}}_{{N_{F} }} \otimes {\text{var}} \left( {\hat{\varvec{g}}_{M} } \right) + {\text{var}} \left( {\hat{\varvec{s}}_{H} } \right)$$

and using ${\text{var}} \left( {\varvec{h}} \right) = {\varvec{W}}^{T} {\varvec{GW}} = {\varvec{G}}_{F} \otimes {\varvec{J}}_{{n_{M} }} + {\varvec{J}}_{{n_{F} }} \otimes {\varvec{G}}_{M} + {\varvec{G}}_{H}$, we get the approximation

26 $$\rho_{a} \left( {\hat{h}_{i \times j} ,h_{i \times j} } \right) \approx \sqrt {\frac{{{\text{var}} \left( {\hat{g}_{F,i} } \right) + {\text{var}} \left( {\hat{g}_{M,j} } \right) + {\text{var}} \left( {\hat{s}_{i \times j} } \right)}}{{{\text{var}} \left( {h_{i \times j} } \right)}}} = \sqrt {\rho_{a}^{2} \left( {\hat{g}_{F,i} ,g_{F,i} } \right)\tau_{{{\text{gca}}F}} + \rho_{a}^{2} \left( {\hat{g}_{M,j} ,g_{M,j} } \right)\tau_{{{\text{gca}}M}} + \rho_{a}^{2} \left( {s_{i \times j} ,\hat{s}_{i \times j} } \right)\tau_{{{\text{sca}}}} }$$

where $$\tau_{{{\text{gca}}F}} = \sigma_{{{\text{gca}}F}}^{2} /\sigma_{G}^{2}$$, $$\tau_{{{\text{gca}}M}} = \sigma_{{{\text{gca}}M}}^{2} /\sigma_{G}^{2}$$ and $$\tau_{{{\text{sca}}}} = \sigma_{{{\text{sca}}}}^{2} /\sigma_{G}^{2}$$.

Following Ould Estaghvirou et al. (2013), an estimate $$\overset{\lower0.5em\hbox{$\smash{\scriptscriptstyle\smile}$}}{r}_{a} \left( \Phi \right)$$ of the prediction accuracy $$r_{a} \left( \Phi \right)$$ of the GBLUPs for the genotypes in a population $$\Phi$$ can be obtained from the (individual) prediction accuracies $$\rho_{a} \left( k \right)$$ of $$k \in \Phi$$ as

27 $$\overset{\lower0.5em\hbox{$\smash{\scriptscriptstyle\smile}$}}{r}_{a} \left( \Phi \right) = \sqrt {\frac{1}{\left| \Phi \right|}\mathop \sum \limits_{k \in \Phi } \rho_{a}^{2} \left( k \right)}$$

In our setting, $\Phi \subset H = \left\{ {\left( {1,1} \right),...,\left( {1,n_{M} } \right),\left( {2,1} \right),...,\left( {2,n_{M} } \right),\left( {n_{F} ,1} \right),...,\left( {n_{F} ,n_{M} } \right)} \right\}$ describes a subset of hybrids. Regarding the prediction accuracy of GBLUPs for H0 hybrids, we have $\Phi_{0,0} = F_{0} \times M_{0}$; for H1F and H1M hybrids, we have $\Phi_{1,0} = F_{1} \times M_{0}$ and $\Phi_{0,1} = F_{0} \times M_{1}$, respectively; and for H2 hybrids we have $\Phi_{1,1} = \left( {F_{1} \times M_{1} } \right)\backslash HT$. Thus, we obtain for H0 and H1 hybrids.

$$\overset{\lower0.5em\hbox{$\smash{\scriptscriptstyle\smile}$}}{r}_{a} \left( {\hat{\varvec{h}}_{{\Phi_{s,t} }} } \right) = \sqrt {\frac{1}{{\left| {F_{a} } \right| \cdot \left| {M_{b} } \right|}}\mathop \sum \limits_{{\left( {i,j} \right) \in \Phi_{s,t} }} \rho_{a}^{2} \left( {\hat{h}_{i \times j} ,h_{i \times j} } \right)}$$

.

Together with Eq. (26), we get

$$\overset{\lower0.5em\hbox{$\smash{\scriptscriptstyle\smile}$}}{r}_{a} \left( {\hat{\varvec{h}}_{{\Phi_{s,t} }} } \right) \approx \sqrt {\frac{1}{{\left| {F_{s} } \right|}}\mathop \sum \limits_{{i \in F_{s} }} \rho_{a}^{2} \left( {\hat{g}_{F,i} ,g_{F,i} } \right)\tau_{{{\text{gca}}F}} + \frac{1}{{\left| {M_{s} } \right|}}\mathop \sum \limits_{{i \in M_{s} }} \rho_{a}^{2} \left( {\hat{g}_{M,j} ,g_{M,j} } \right)\tau_{{{\text{gca}}M}} + \frac{1}{{\left| {F_{s} } \right| \cdot \left| {M_{t} } \right|}}\mathop \sum \limits_{{\left( {i,j} \right) \in F_{a} \times M_{b} }} \rho_{a}^{2} \left( {\hat{s}_{i \times j} ,s_{i \times j} } \right)\tau_{{{\text{sca}}}} }$$

From Eq. (27), we have $$r_{a} \left( {\hat{\varvec{g}}_{{F,F_{s} }} } \right) \approx \overset{\lower0.5em\hbox{$\smash{\scriptscriptstyle\smile}$}}{r}_{a} \left( {\hat{\varvec{g}}_{{F,F_{s} }} } \right) = \sqrt {\frac{1}{{\left| {F_{s} } \right|}}\mathop \sum \nolimits_{{i \in F_{s} }} \rho_{a}^{2} \left( {\hat{g}_{F,i} ,g_{F,i} } \right)}$$,

$$r_{a} \left( {\hat{\varvec{g}}_{{M,M_{t} }} } \right) \approx \overset{\lower0.5em\hbox{$\smash{\scriptscriptstyle\smile}$}}{r}_{a} \left( {\hat{\varvec{g}}_{{M,M_{t} }} } \right) = \sqrt {\frac{1}{{\left| {M_{t} } \right|}}\mathop \sum \nolimits_{{j \in M_{t} }} \rho_{a}^{2} \left( {\hat{g}_{{M,M_{t} }} ,g_{{M,M_{t} }} } \right)}$$

and $$r_{a} \left( {\hat{\varvec{s}}_{{F_{s} \times M_{t} }} } \right) \approx \overset{\lower0.5em\hbox{$\smash{\scriptscriptstyle\smile}$}}{r}_{a} \left( {\hat{\varvec{s}}_{{F_{s} \times M_{t} }} } \right) = \sqrt {\frac{1}{{\left| {F_{s} } \right| \cdot \left| {M_{t} } \right|}}\mathop \sum \nolimits_{{\left( {i,j} \right) \in F_{s} \times M_{t} }} \rho_{a}^{2} \left( {\hat{s}_{i \times j} ,s_{i \times j} } \right)}$$.

Combining these results, we get as an approximation for $$r_{a} \left( {\hat{\varvec{h}}_{{F_{s} \times M_{t} }} } \right) \approx { }\overset{\lower0.5em\hbox{$\smash{\scriptscriptstyle\smile}$}}{r}_{a} \left( {\hat{\varvec{h}}_{{\Phi_{s,t} }} } \right)$$

28 $$r_{a} \left( {\hat{\varvec{h}}_{{F_{s} \times M_{t} }} } \right) \approx \sqrt {r_{a}^{2} \left( {\hat{\varvec{g}}_{{F,F_{s} }} } \right)\tau_{{{\text{gca}}F}} + r_{a}^{2} \left( {\hat{\varvec{g}}_{{M,M_{t} }} } \right)\tau_{{{\text{gca}}M}} + r_{a}^{2} \left( {\hat{\varvec{s}}_{{F_{s} \times M_{t} }} } \right)\tau_{{{\text{sca}}}} }$$

Substituting $r_{a} \left( {\hat{\varvec{g}}_{{F,F_{s} }} } \right)$, $\tau_{{{\text{gca}}F}}$ and the other terms on the right hand side by estimates such as $\hat{r}_{a} \left( {\hat{\varvec{g}}_{{F,F_{s} }} } \right)$ etc. yields the estimate $\tilde{r}_{a} \left( {\hat{\varvec{h}}_{{F_{s} \times M_{t} }} } \right)$ for $r_{a} \left( {\hat{\varvec{h}}_{{F_{s} \times M_{t} }} } \right)$:

29 $$\tilde{r}_{a} \left( {\hat{\varvec{h}}_{{F_{s} \times M_{t} }} } \right) = \sqrt {\hat{r}_{a}^{2} \left( {\hat{\varvec{g}}_{{F,F_{s} }} } \right)\tau_{{{\text{gca}}F}} + \hat{r}_{a}^{2} \left( {\hat{\varvec{g}}_{{M,M_{t} }} } \right)\tau_{{{\text{gca}}M}} + \hat{r}_{a}^{2} \left( {\hat{\varvec{s}}_{{F_{s} \times M_{t} }} } \right)\tau_{{{\text{sca}}}} }$$

which was used for preparing Figures S4 and S6.

If the TS is constructed according to a balanced incomplete factorial design with $c$ crosses per parent line in $F_{1}$ and $M_{1}$ and $\left| {F_{1} } \right| = \left| {M_{1} } \right|$, using Eqs. (26 and 27) we have for H2 hybrids

$$\begin{gathered} \tilde{r}_{a} \left( {\hat{\user2{h}}_{{\Phi_{1,1} }} } \right) = \sqrt {\frac{1}{{\left| {F_{1} } \right| \cdot \left( {\left| {M_{1} } \right| - c} \right)}}\mathop \sum \limits_{{\left( {i,j} \right) \in \Phi_{1,1} }} \rho_{a}^{2} \left( {\hat{h}_{i \times j} ,h_{i \times j} } \right)} \approx \sqrt {\frac{1}{{\left| {F_{1} } \right|}}\mathop \sum \limits_{{i \in F_{1} }} \rho_{a}^{2} \left( {\hat{g}_{F,i} ,g_{F,i} } \right)\tau_{{{\text{gca}}F}} + \frac{1}{{\left| {M_{1} } \right|}}\mathop \sum \limits_{{j \in M_{1} }} \rho_{a}^{2} \left( {\hat{g}_{M,j} ,g_{M,j} } \right)\tau_{{{\text{gca}}M}} + \frac{1}{{\left| {F_{1} } \right| \cdot \left( {\left| {M_{1} } \right| - c} \right)}}\mathop \sum \limits_{{\left( {i,j} \right) \in \Phi_{1,1} }} \rho_{a}^{2} \left( {\hat{s}_{i \times j} ,s_{i \times j} } \right)\tau_{{{\text{sca}}}} } \hfill \\ \sqrt {\frac{1}{{\left| {F_{1} } \right|}}\mathop \sum \limits_{{i \in F_{1} }} \rho_{a}^{2} \left( {\hat{g}_{F,i} ,g_{F,i} } \right)\tau_{{{\text{gca}}F}} + \frac{1}{{\left| {M_{1} } \right|}}\mathop \sum \limits_{{j \in M_{1} }} \rho_{a}^{2} \left( {\hat{g}_{M,j} ,g_{M,j} } \right)\tau_{{{\text{gca}}M}} + \frac{1}{{\left| {F_{1} } \right| \cdot \left( {\left| {M_{1} } \right| - c} \right)}}\mathop \sum \limits_{{\left( {i,j} \right) \in \Phi_{1,1} }} \rho_{a}^{2} \left( {\hat{s}_{i \times j} ,s_{i \times j} } \right)\tau_{{{\text{sca}}}} } \hfill \\ \end{gathered}$$

Using again Eq. (28), we get

30 $$\tilde{r}_{a} \left( {\hat{\varvec{h}}_{{\Phi_{1,1} }} } \right) \approx \sqrt {r_{a}^{2} \left( {\hat{\varvec{g}}_{{F,F_{1} }} } \right)\tau_{{{\text{gca}}F}} + r_{a}^{2} \left( {\hat{\varvec{g}}_{{M,M_{1} }} } \right)\tau_{{{\text{gca}}M}} + r_{a}^{2} \left( {\hat{\varvec{s}}_{{\Phi_{1,1} }} } \right)\tau_{{{\text{sca}}}} }$$

as an approximation of $r_{a} \left( {\hat{\varvec{h}}_{{\Phi_{1,1} }} } \right)$.

In summary, Eqs. (29 and 30) allow to estimate $\tilde{r}_{a}$ for the prediction accuracy $r_{a}$ of H0 hybrids $\left( {\Phi_{0,0} = F_{0} \times M_{0} } \right)$, H1 hybrids ($\Phi_{1,0} = F_{1} \times M_{0}$ or $\Phi_{0,1} = F_{0} \times M_{1}$) and H2 hybrids ($\Phi_{1,1} = \left( {F_{1} \times M_{1} } \right)\backslash HT$) by substituting appropriate $r_{a}$ estimates of their I0 and/or I1 parent lines and $r_{a}$ estimates of the SCA effects of their hybrids.
