# Supplementary material for: Genomic prediction in hybrid breeding: I. Optimizing the training set design
Source: Theor Appl Genet. 2023 Aug 2;136(8):176. doi: 10.1007/s00122-023-04413-y (PMC10397156; doi:10.1007/s00122-023-04413-y)
Supplement: Supplementary file 1 — Supplementary file1 (PDF 1218 KB) [file 122_2023_4413_MOESM1_ESM.pdf]

Supplement of Genomic prediction in hybrid breeding: I. Optimizing the training set design

Table S1. Scenarios used for simulating traits differing in $\tau_{sca} = 100 \times \sigma^2_{sca}:\sigma^2_G$ (the proportion of SCA variance in the total genetic variance σ^2_G of hybrids in set H) for data set DS1 and DS2. n_Q refers to the total number of QTL, n_{Q_d} to the number of QTL with only dominance effects. Additive effects were sampled from a Gamma distribution with parameter scale = 1.66 and shape = 0.4. The degree of dominance k_i was sampled from $k_i \sim N(\mu_k, \sigma^2_k)$. $\bar{\tau}_{sca}$ ± SD refer to the mean and standard deviation of τ_{sca} over 50 simulation runs for each trait (for details see text). h^2 refers to the heritability of the hybrids in H.

"Target" trait	Scenario $h^2 = 0.4$	Scenario $h^2 = 0.8$	n_Q DS1/DS2	n_{Q_d} DS1/DS2	μ_k	σ_k	Data set DS1 $\bar{\tau}_{sca}$ ± SD	Data set DS2 $\bar{\tau}_{sca}$ ± SD
Yield	1	2	1000/500	500/250	1.00	0.33	22 ± 22	22 ± 7
Maturity	3	4	1000/500	0/0	0.50	0.15	6 ± 7	6 ± 2
Quality	5	6	300/200	0/0	0.18	0.06	1 ± 2	1 ± 0.3

Table S2. Estimates of general combining ability (GCA, $\boldsymbol{\sigma^2_{gcaF}}$, $\boldsymbol{\sigma^2_{gcaM}}$) and specific combining ability (SCA, $\boldsymbol{\sigma^2_{sca}}$) variances and proportion $\boldsymbol{\tau_{sca}} = \frac{100\times\sigma^2_{sca}}{\sigma^2_{gcaF}+\sigma^2_{gcaM}+\sigma^2_{sca}}$ in the hybrid population between two parent populations $F$and$M$ for agronomic traits reported in the maize literature.

Parent pops. F and M	Trait	$\boldsymbol{\sigma^2_{gcaF}}$	$\boldsymbol{\sigma^2_{gcaM}}$	$\boldsymbol{\sigma^2_{sca}}$	$\boldsymbol{\tau_{sca}}$ (in %)§	Source
BSSS, BSCB1 maize	Grain yield	0.137	0.147	0.117	30.0	Betran and Hallauer (1996)
	Grain moisture	1.223	1.270	0.240	8.8	Keeratinijakal and Lamkey (1993)
	Plant height	53.89	48.83	10.9	9.5	
BSSS, NSSS maize	Grain yield	0.42	0.29	0.09	11	Kadam et al. (2016)
	Plant height	78.82	86.34	12.30	6.8	
Euro. Dent, Flint maize	Grain yield	32.79	28.12	8.44	12.1	Technow et al. (2014)
	Grain moisture	2.58	2.59	0.40	7.18	
Euro. Dent, Flint maize	Dry matter yield	1.51	1.00	0.17	6.34	Westhues et al. (2017)
	Dry matter cont.	4.17	5.03	0.49	5.04	
	Protein content	3.11	2.77	0.29	4.02	
Euro. Dent, Flint maize	Dry matter yield	0.30	0.44	0.20	21.0	Giraud et al. (2017)
	Flowering time	0.51	1.09	0.47	22.6	
	Plant height	38.4	45.1	13.4	12.4	

References

Betran F, Hallauer A (1996) Characterization of interpopulation genetic variability in three hybrid maize populations. Journal of Heredity 87:319-328

Giraud H, Bauland C, Falque M, Madur D, Combes V, Jamin P, Monteil C, Laborde J, Palaffre C, Gaillard A (2017) Reciprocal genetics: identifying QTL for general and specific combining abilities in hybrids between multiparental populations from two maize (Zea mays L.) heterotic groups. Genetics 207:1167-1180

Kadam DC, Potts SM, Bohn MO, Lipka AE, Lorenz AJ (2016) Genomic prediction of single crosses in the early stages of a maize hybrid breeding pipeline. G3: Genes, Genomes, Genetics 6:3443-3453

Keeratinijakal V, Lamkey KR (1993) Responses to reciprocal recurrent selection in BSSS and BSCB1 maize populations. Crop Science 33:73-77

Technow F, Schrag TA, Schipprack W, Bauer E, Simianer H, Melchinger AE (2014) Genome properties and prospects of genomic prediction of hybrid performance in a breeding program of maize. Genetics 197:1343-1355

Westhues M, Schrag TA, Heuer C, Thaller G, Utz HF, Schipprack W, Thiemann A, Seifert F, Ehret A, Schlereth A (2017) Omics-based hybrid prediction in maize. Theoretical and applied genetics 130:1927-1939

Supplement of Genomic prediction in hybrid breeding: I. Optimizing the training set design

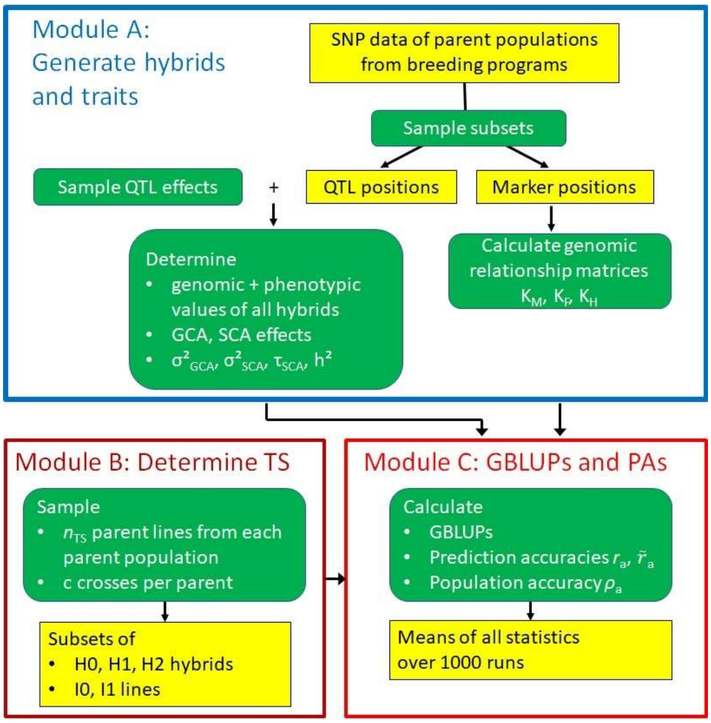

Figure S1. Flow chart representing the various steps involved in simulating the genotypic and phenotypic data of the different traits (Module A), training and prediction sets (Module B), and genomic prediction with GBLUP and analyses of the results (Module C). Angular boxes refer to the generated data and round boxes to the applied operations.

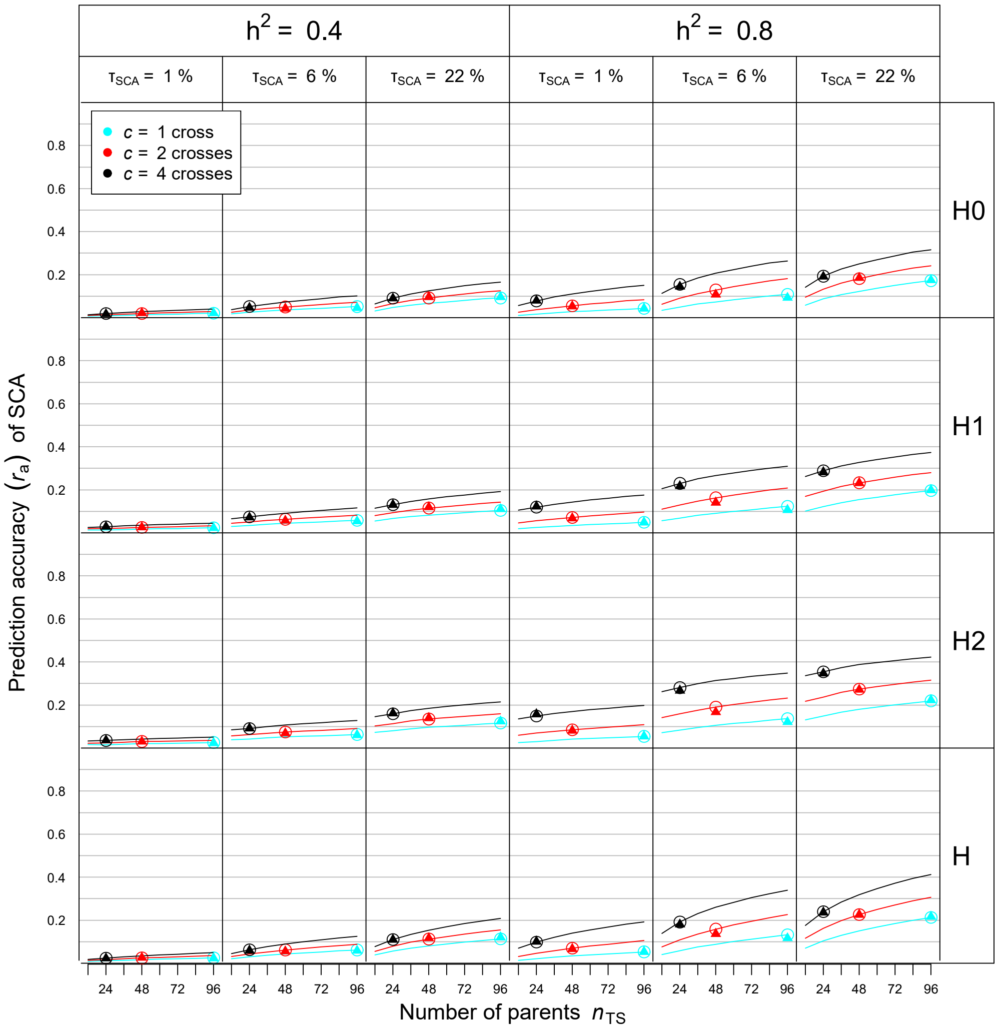

Figure S2. Prediction accuracy (r_a) for SCA of H0, H1 and H2 types of hybrids and all hybrids in set H as a function of the number n_{TS} of parent lines and number of crosses per parent ($c = 1, 2, 4$) used for producing the training set (TS). Results refer to means over 1,000 simulation runs based on data set DS1 for different values of h^2 and τ_{SCA}. Circles and triangles refer to r_a values for $N_{TS} = n_{TS} \times c = 96$ obtained with GBLUPs calculated with "true" and estimated variance components, respectively.

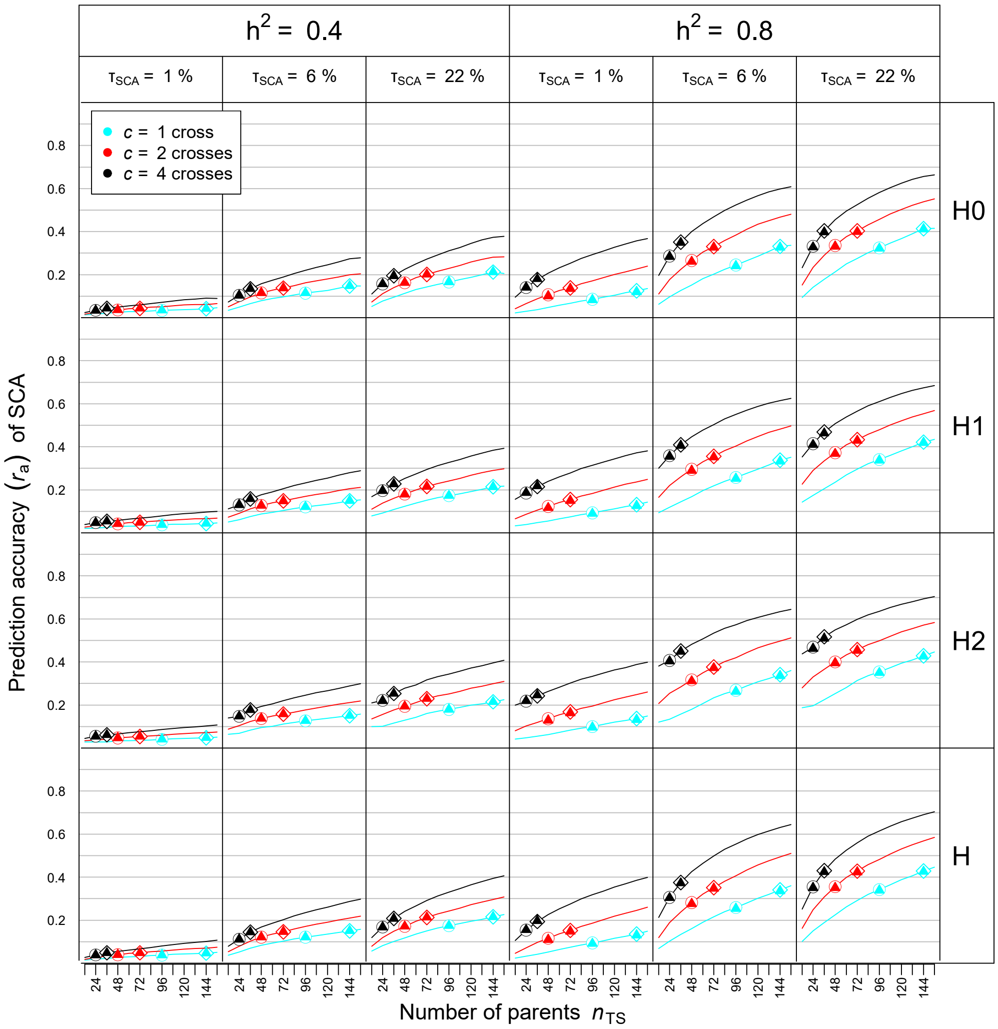

Figure S3. Prediction accuracy (r_a) for SCA of H0, H1 and H2 types of hybrids and all hybrids in set H as a function of the number n_{TS} of parent lines and number of crosses per parent ($c = 1, 2, 4$) used for producing the training set (TS). Results refer to means over 1,000 simulation runs based on data set DS2 for different values of h^2 and τ_{SCA}. Circles and triangles refer to results for $N_{TS} = n_{TS} \times c = 96$ and diamonds and triangles refer to results for $N_{TS} = n_{TS} \times c = 144$ obtained with GBLUPs calculated with "true" and estimated variance components, respectively.

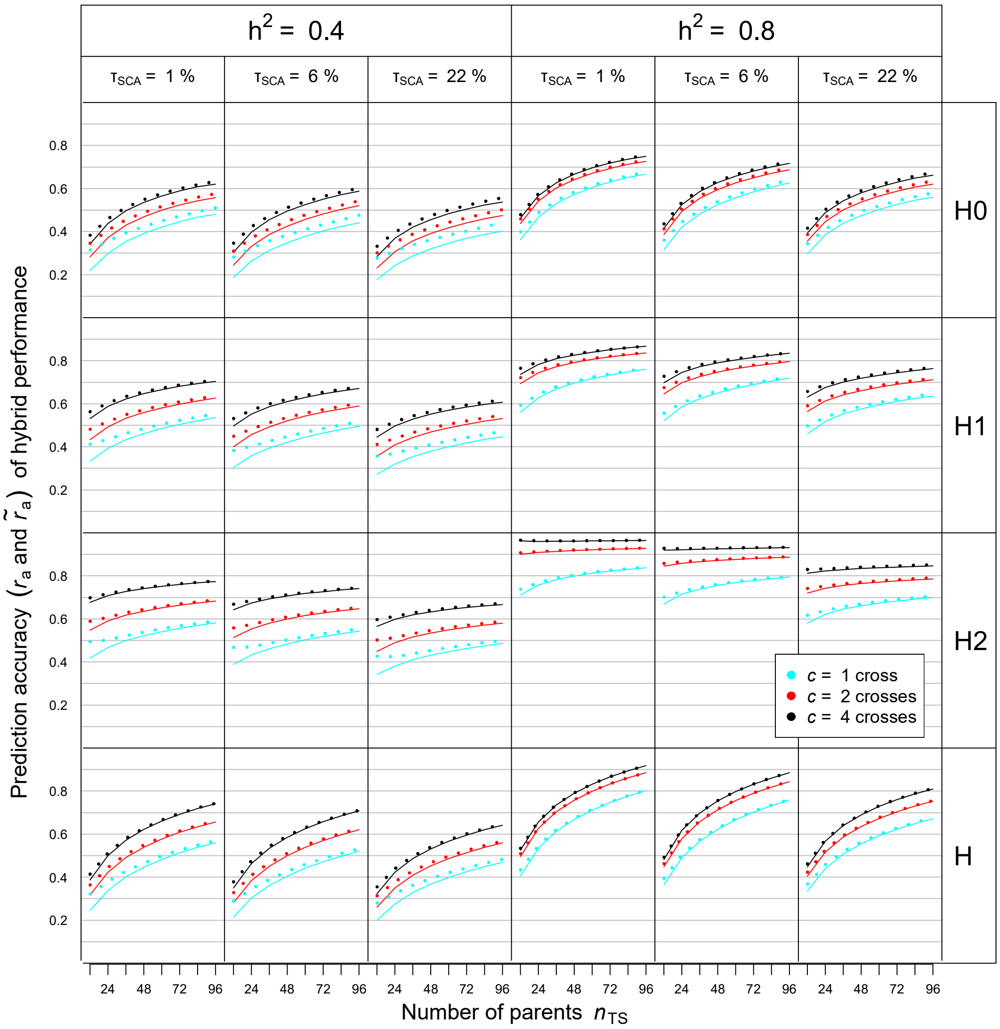

Figure S4. Approximated prediction accuracy ($\tilde{r}_a$, dotted curves calculated according to Eqn. (16)) for (A) H0, H1, and H2 type of hybrids and all hybrids in set H and (B) GCA of I0 and I1 lines as a function of the number $n_{TS}$of parent lines and number of crosses per parent ($c = 1, 2, 4$) used for producing the training set (TS). The r_a values (solide curves) shown in Figure 2 are included for comparison. Results refer to means of 1,000 simulation runs based on data set DS1 for different values of h^2 and τ_{SCA} (proportion of the SCA variance in σ_g^2 of hybrids).

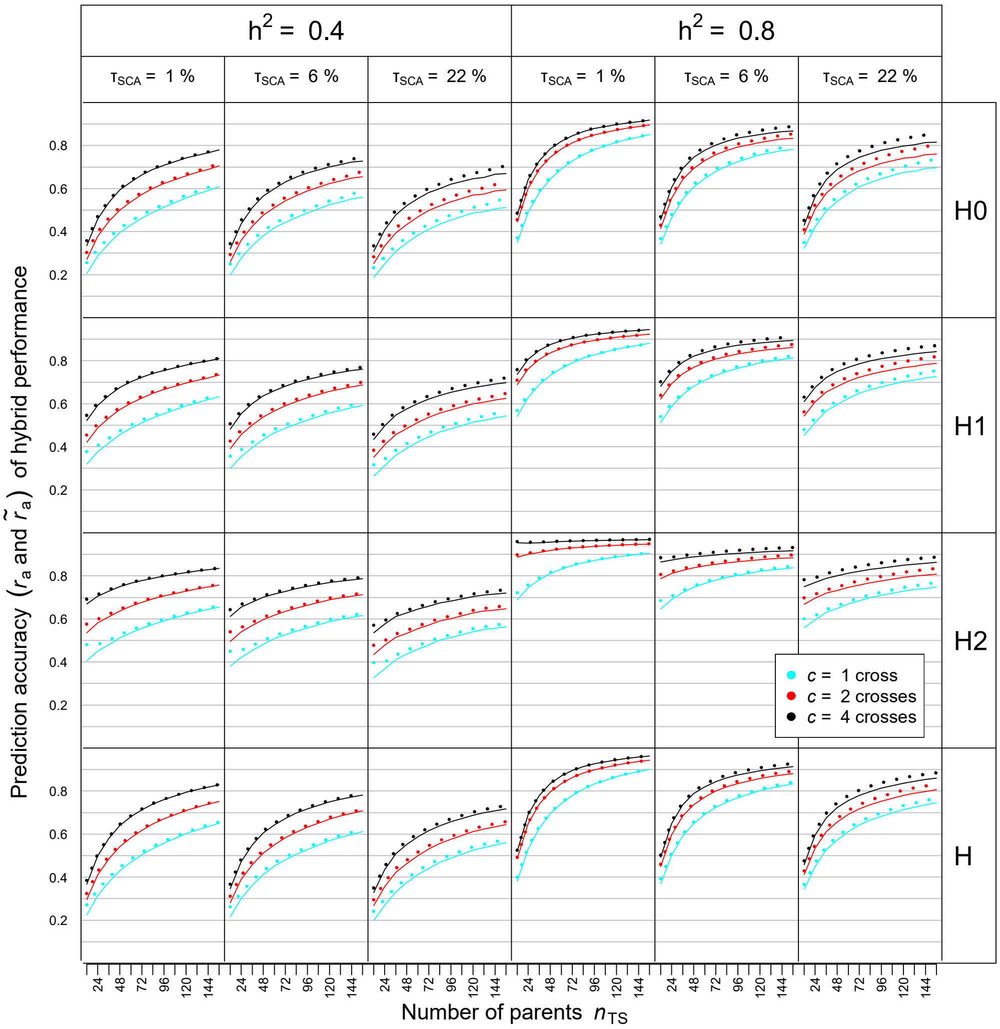

Figure S5. Approximated prediction accuracy ($\tilde{r}_a$, dotted curves calculated according to Eqn. (16)) for (A) H0, H1, and H2 type of hybrids and all hybrids in set H and (B) GCA of I0 and I1 lines as a function of the number n_{TS} of parent lines and number of crosses per parent ($c = 1, 2, 4$) used for producing the training set (TS). The r_a values (solide curves) shown in Figure 3 are included for comparison. Results refer to means of 1,000 simulation runs based on data set DS2 for different values of h^2 and τ_{SCA}.

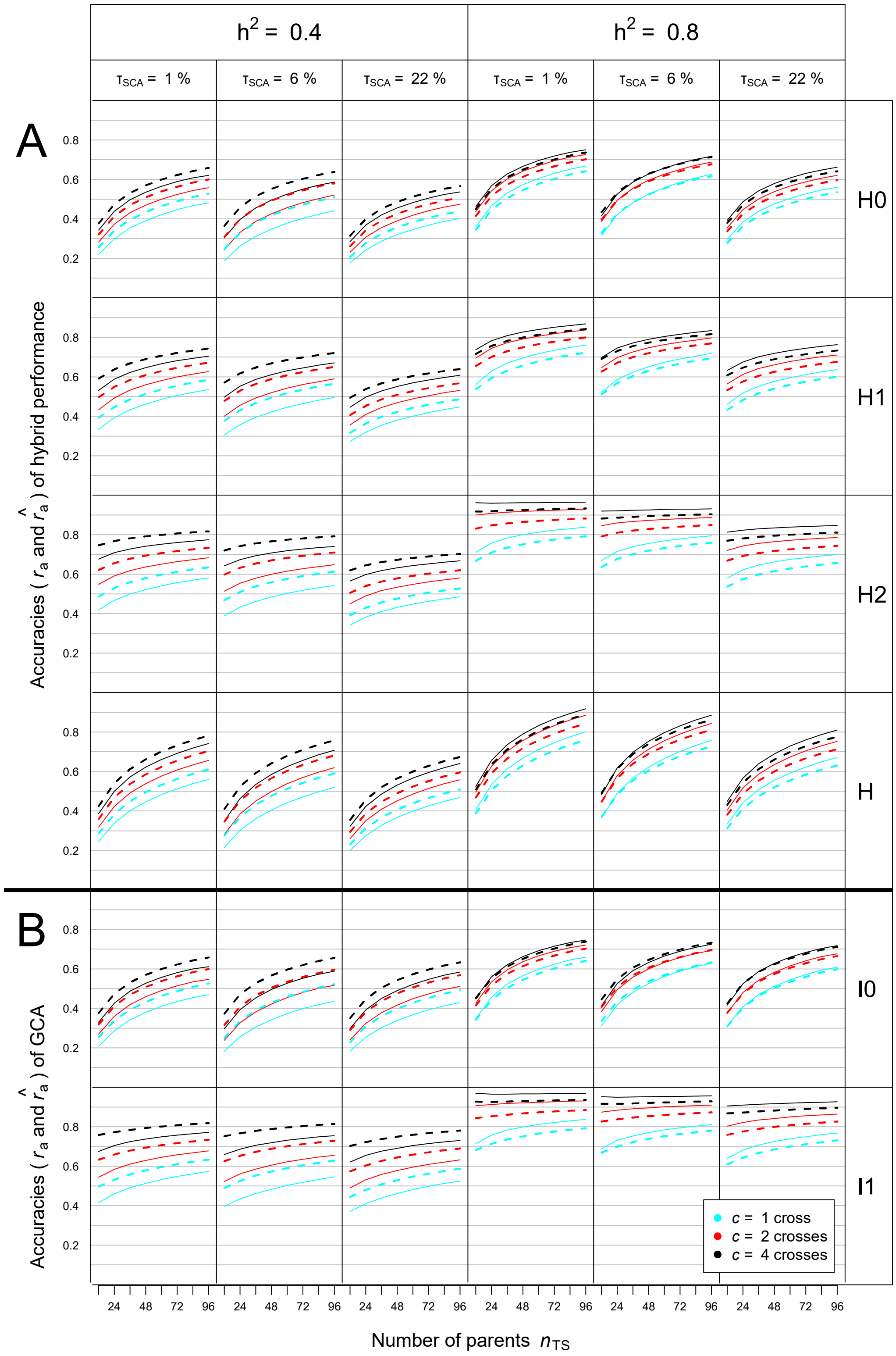

Figure S6. Approximated expectation of the prediction accuracy ($\hat{r}_a$, dashed curves calculated according to equation Eqns. (13, 14)) for (A) H0, H1, and H2 type of hybrids and all hybrids in set H and (B) GCA of I0 and I1 lines as a function of the number n_{TS} of parent lines and number of crosses per parent ($c = 1, 2, 4$) used for producing the training set (TS). The r_a values (solide curves) shown in Figure 2 are included for comparison. Results refer to means of 1,000 simulation runs based on data set DS1 for different values of h^2 and τ_{SCA}.

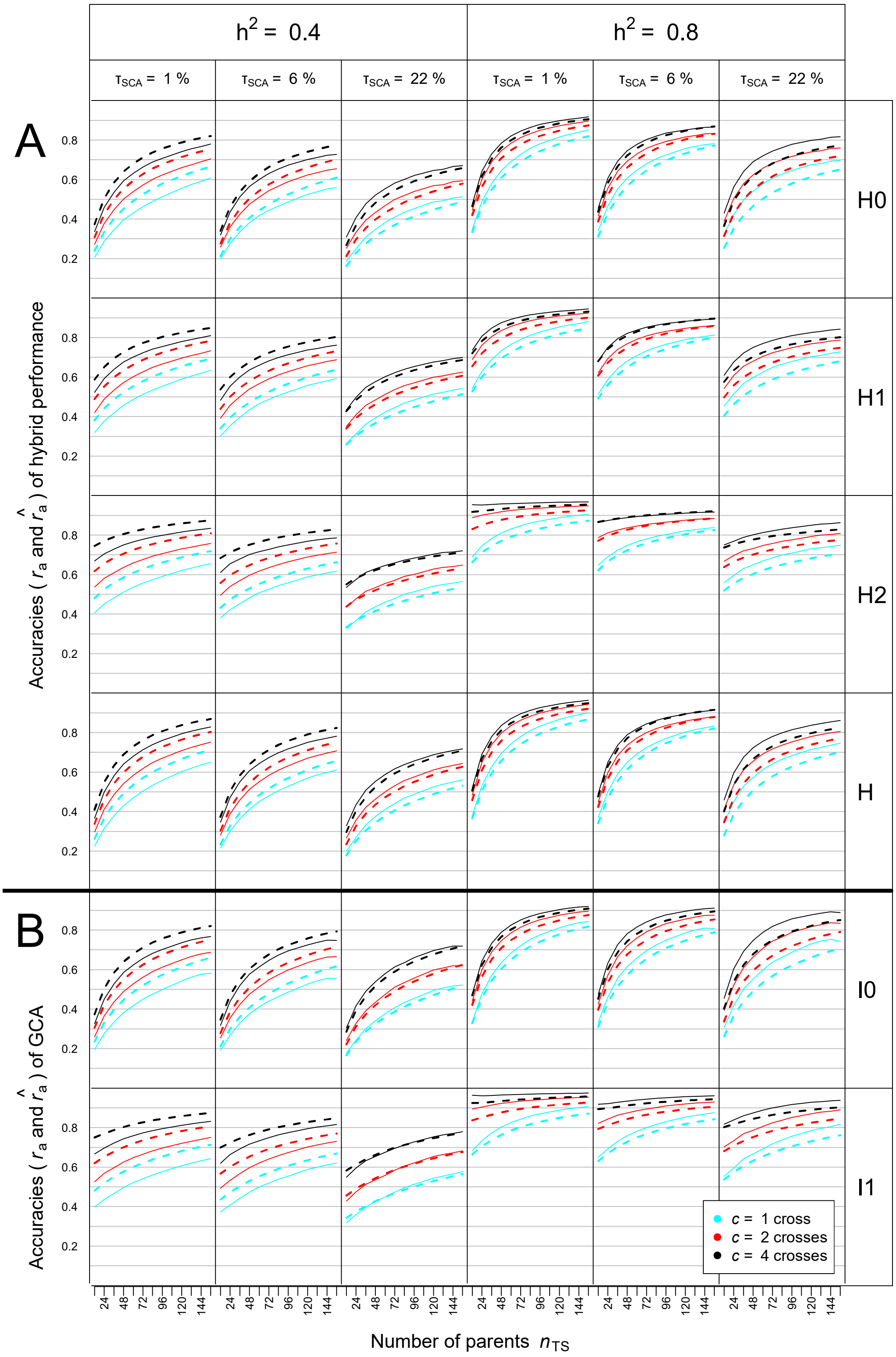

Figure S7. Approximated expectation of the prediction accuracy ($\hat{r}_a$, dashed curves calculated according to equation Eqns. (13, 14)) for (A) H0, H1, and H2 type of hybrids and all hybrids in set H and (B) GCA of I0 and I1 lines as a function of the number n_{TS} of parent lines and number of crosses per parent ($c = 1, 2, 4$) used for producing the training set (TS). The r_a values (solide curves) shown in Figure 3 are included for comparison. Results refer to means of 1,000 simulation runs based on data set DS2 for different values of h^2 and τ_{SCA}.
